# Efficacy of acupuncture therapy on cancer-related insomnia: a systematic review and network meta-analysis

**DOI:** 10.3389/fneur.2024.1342383

**Published:** 2024-02-13

**Authors:** Liying Chen, Jiaqi Li, Shiting Xu, Zhiyi Liu, Yang Jiao, Zhongyu Zhou

**Affiliations:** ^1^Hubei Provincial Hospital of Traditional Chinese Medicine, Wuhan, China; ^2^Affiliated Hospital of Hubei University of Chinese Medicine, Wuhan, China; ^3^Optics Valley Hospital District Medical Office, Hospital of Stomatology Wuhan University, Wuhan, China; ^4^College of Traditional Chinese Medicine, Hubei University of Chinese Medicine, Wuhan, China

**Keywords:** network meta-analysis, cancer-related insomnia, the complication of cancer, acupuncture therapy, non-pharmacological therapy

## Abstract

**Objectives:**

Cancer-related insomnia (CRI) takes a toll on many cancer survivors, causing distressing symptoms and deteriorating the quality of life. Acupuncture therapy has been used for CRI already. However, it is still uncertain which acupuncture regime is best for CRI. The primary objective of this review is to conduct a comparative evaluation and ranking of the effectiveness of different acupuncture therapies for CRI.

**Methods:**

Randomized controlled trials (RCTs) that were published up to July 31, 2023, from 8 databases (PubMed, Embase, Cochrane library, Web of Science, China National Knowledge Infrastructure, Wanfang Database, VIP Database, and China Biology Medicine disc) were integrated in this study. Trials that met the inclusion criteria were evaluated the risk of bias. Pittsburgh sleep quality index (PSQI) was used to assess the efficacy of different acupuncture therapies as the primary outcome. Then, STATA 15, R, and OpenBUGS were applied to perform the network meta-analysis. PRISMA statements were followed in this network meta-analysis.

**Results:**

A total of 37 studies were included in this review, involving 16 interventions with 3,246 CRI participants. Auriculotherapy + moxibustion [surface under the cumulative ranking curve (SUCRA) 98.98%] and auriculotherapy (SUCRA 77.47%) came out top of the ranking, which were more effective than control, medicine, usual care and sham acupuncture.

**Conclusion:**

Auriculotherapy + moxibustion and auriculotherapy + acupuncture emerged as the top two acupuncture regimes for CRI and future studies should pay more attention to CRI.

**Clinical trial registration:**

https://clinicaltrials.gov/, identifier INPLASY202210095.

## 1 Introduction

Cancer-related insomnia (CRI) is a substantial complication of cancer, tormenting many cancer survivors. It is manifested as sleep initiation disorder, frequent night awakenings, early awakenings, or dreamy in the process of the diagnosis and treatment of cancer (1–3). These annoying symptoms also cause a sequence of adverse effects on cancer survivors, such as impairment in daytime function and quality of life, anxiety and depression (4, 5). As is reported, 30–50% (up to 95%) of cancer survivors suffer from CRI, and the incidence is exceptionally high in lung cancer and breast cancer (1, 6, 7). Since CRI threatens the quality of life and prognosis of cancer survivors, attention should be paid to the treatment of CRI.

Regarding the treatment of CRI, both pharmacotherapy and non-pharmacological therapies can be applied to relieve CRI symptoms. The medications used to manage CRI are mainly sedatives and melatonin. Although these medications do alleviate sleep disturbances in many cancer survivors with CRI, they may produce many side effects such as withdrawal, cognitive impairment, headache and so on, which leads to poor tolerance and low adherence of medication (3, 8). Cognitive behavioral therapy (CBT), as a non-pharmacological therapy recommended in guidelines for various types of insomnia (9, 10), has been confirmed to have a definite curative effect on CRI (11, 12). But the popularizing rate of CBT was not so desirable due to its time-consuming, complicated, and more effort and self-discipline required than normal therapy (13). Acupuncture therapy is effective, low risky, and easy to implement for the treatment of CRI, so increasing cancer survivors are turning to the assistance of acupuncture therapy (14–16). Acupuncture therapy encompasses but is not limited to acupuncture, electroacupuncture, auriculotherapy, moxibustion and acupoint application. A brief introduction of conventional acupuncture was shown in Supplementary Figures S1–S6.

A considerable number of relative randomized controlled trials have been executed to authenticate the efficacy of acupuncture therapy in treating CRI. Acupuncture therapy could improve habitual sleep efficiency, prolong sleep duration, reduce the frequency of sleep awakenings (17, 18). In recent years, meta-analyses have also corroborated the effectiveness of acupuncture therapies in treating CRI. Studies (19, 20) have demonstrated that acupuncture therapy could effectively ameliorate sleep disorders, augment the sleep duration and reduce the reliance on sleep medication in CRI patients. However, above meta-analyses only compared acupuncture therapy in pairs. Ou et al. (21) compared the efficacy of different acupuncture therapies on CRI, but we noticed that several studies included in the network meta-analysis were related to surgery. Consequently, it was impossible to define whether insomnia was cancer-related or surgery-related in these studies. Moreover, several studies within the network meta-analysis incorporated other non-pharmacological interventions in the experimental group, which may impact the ranking of acupuncture therapies. Therefore, which acupuncture regime has the first-class curative effect is still in the mist and the development of acupuncture therapy for CRI is a need in the field. Thus, the dominating purpose of this review is to compare the difference of the curative effect among acupuncture regimens for CRI and rank the efficacy of acupuncture therapies for CRI.

## 2 Methods

### 2.1 Study design

This network meta-analysis adhered to the Preferred Reporting Items for Systematic Reviews and Meta-Analyses (PRISMA) guidelines published in 2020 and procedures shown in the Cochrane Handbook. The protocol for this network meta-analysis has been published (22). Informed consent was not required as the data used in this review derived from pre-existing articles.

### 2.2 Eligible criteria

#### 2.2.1 Patients

Cancer survivors with CRI.

#### 2.2.2 Intervention

Acupuncture therapies include but not limited to acupuncture, moxibustion, electroacupuncture, acupressure, auriculotherapy and acupoint application. Pharmacotherapy and usual care could be combined with the above-mentioned acupuncture therapies.

#### 2.2.3 Comparator

Medications, usual care and sham acupuncture interventions.

#### 2.2.4 Outcome

In this study, Pittsburgh sleep quality index (PSQI) was used to assess the efficacy of different acupuncture therapies as the primary outcome, with the secondary outcomes being the subitems of the PSQI.

#### 2.2.5 Study design

Only peer-reviewed randomized controlled trials with available and detailed data were included.

### 2.3 Exclusion criteria

(1) Reviews, animal experiments, case reports, protocols, systematic reviews, conference papers, comments, etc. (2) Primary insomnia or other secondary insomnia. (3) Non-randomized controlled trials such as inappropriate random method, retrospective study, etc. (4) Other complementary or alternative therapies were included in experimental group and/or control group, which are not limited to psychological intervention, behavioral therapy, herbal therapy, etc. (5) Identical acupuncture therapy with varying treatment time or materials. (6) Duplicate published literature or literature without full text or specific data.

### 2.4 Search strategy

A full-scale electronic literature search was conducted across 8 databases (PubMed, Embase, Cochrane library, Web of Science, China National Knowledge Infrastructure, Wanfang Database, VIP Database, and China Biology Medicine disc) to retrieve English and Chinese literature from setup until 31st July 2023. Based on the retrieval needs of each database, we personalized the search strategy, and the detailed search strategies were displayed in Supplementary Tables S1–S8.

### 2.5 Data extraction

Two reviewers screened titles and abstracts severally to assess eligible RCTs using EndNote X9 software. Subsequently, a comprehensive review was performed with the full-text of included articles for further evaluation and data extraction. The data to be extracted were studies’ characteristics (author, year of publication, country), participants’ characteristics (sample size, types of acupuncture therapies, and comparisons), and results. Any discrepancies or disagreements between two reviewers were adjudicated by a third reviewer.

### 2.6 Risk of bias

The risk of bias of included studies was performed based on Cochrane risk-of-bias tool (ROB 2.0) (23) and was classified into “low risk,” “high risk,” or “some concern.” In the event of any discrepancies, a third investigator was consulted for adjudication.

Bias was evaluated by reviewers in the following five domains: (1) bias generating during randomization, (2) bias due to deviations from expected interventions, (3) bias from missing outcome data, (4) bias in outcome measures, (5) bias in the selection of the reported outcomes.

### 2.7 Data synthesis and statistical methods

Network meta-analyses were carried out by OpenBUGS, STATA 15, and R. Global consistency was performed by residual deviance and local consistency by node spilt analysis. Then, OpenBUGS analyzed MD and CI for each outcome with followed parameters: number of chain = 3, tuning iterations = 25,001, thinning interval = 1, stimulation iterations = 100,000. In addition, MD and CI were transformed into the surface under the cumulative ranking curves (SUCRA) and league figures by STATA 15 to visualize the comparisons. Funnel plots were applied to evaluate potential publication bias. Moreover, subgroup and sensitivity analysis would be carried out if required.

## 3 Results

### 3.1 Study identification and selection

**Figure 1:**
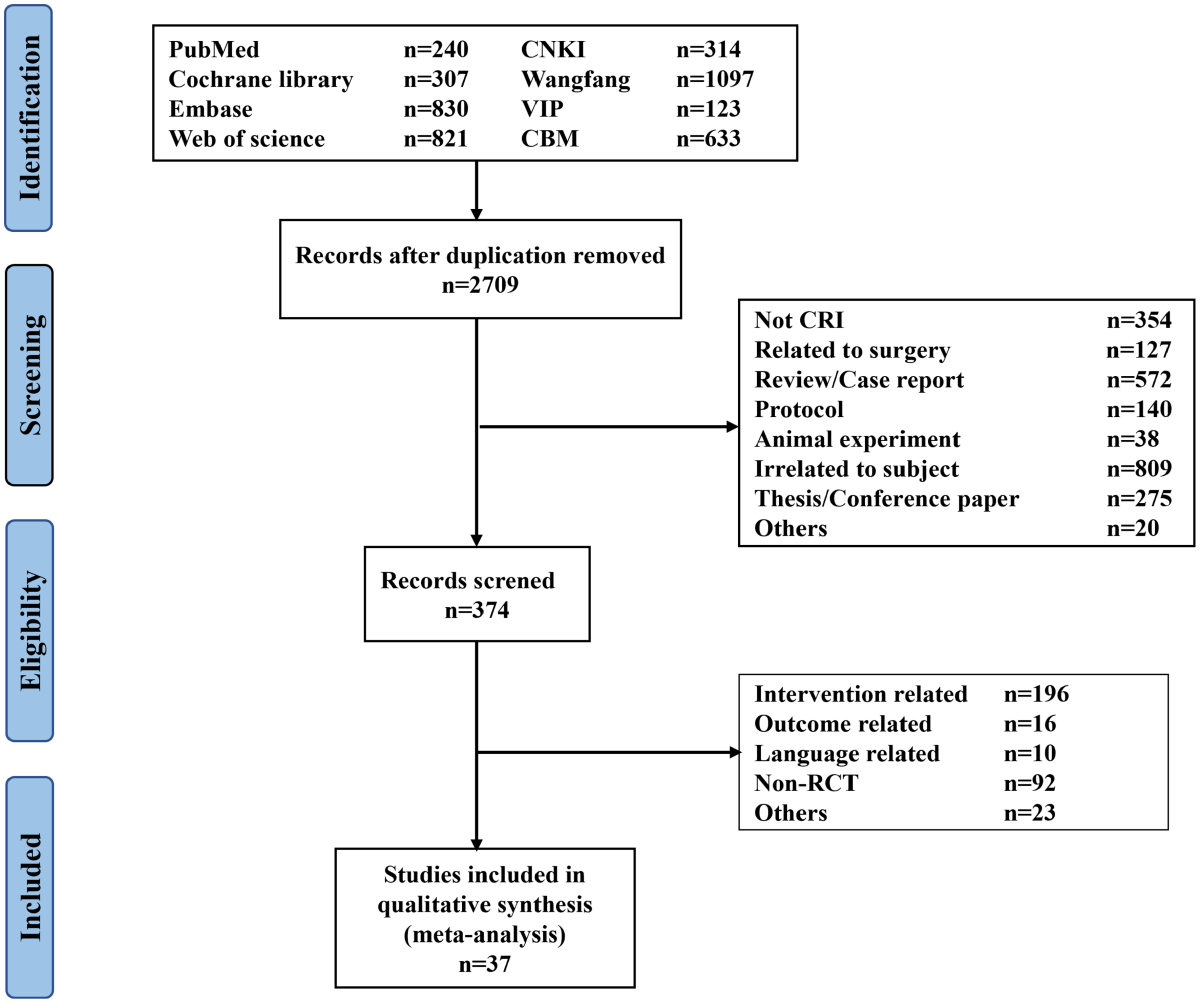
PRISMA flow diagram of the study selection process. CNKI, China National Knowledge Infrastructure; VIP, China Science and Technology Journal Database.

A comprehensive search of eight electronic databases yielded a total of 4,365 relevant studies. After eliminating duplicate studies, 2,335 studies were discarded through examination of titles and abstracts, and 374 articles proceeded to the full-text review stage. Subsequently, 337 studies were ruled out following a thorough assessment of their full text. Ultimately, 37 studies satisfied the inclusion criteria. Then, R was applied to perform node spilt analysis for each outcome and all outcomes met the requirement of consistency (Supplementary Figures S7–S13). Consequently, 37 studies were eventually incorporated into this network meta-analysis. Figure 1 showed the selection process for this review.

### 3.2 Description of included studies

The 37 studies included 35 two-arm trials and two multi-arm trials, involving of 3,246 participants and 16 interventions which contain 10 single interventions and six combined interventions. Among the included studies, 28 studies have been published within the past 5 years, indicating that acupuncture therapy on CRI is garnering increasing attention. The characteristics of eligible studies were presented in Table 1. The usage statistics of acupoints in included studies was displayed in Supplementary Table S9. And the most commonly employed acupoints on CRI were Xin (heart, CO15) and Shenmen (TF4).

**Table 1:** Characteristics of included studies.

Id	Study	Year	Region/Language	n	Intervention (control group—experimental group)
1	Feng, Y	2011	China/English	80	Medicine − acupuncture
2	Hyeon GY	2019	Korea/English	41	Sham − auriculotherapy
3	Lee B	2022	Korea/English	22	Usual care − sham − electroacupuncture
4	Wang Y	2022	China/English	68	Control − auriculotherapy
5	Zhang J	2023	China/English	117	Sham − acupuncture
6	Zhang J	2021	China/Chinese	28	Control – auriculotherapy + electroacupuncture
7	Cao M	2020	China/Chinese	80	Sham-acupoint application
8	Chen SY	2021	China/Chinese	64	Medicine – auriculotherapy +acupuncture
9	Chen YL	2022	China/Chinese	78	Usual care – auriculotherapy
10	Fa XY	2021	China/Chinese	80	Control – auriculotherapy + acupoint application
11	Fan GH	2020	China/Chinese	97	Medicine – auriculotherapy + acupuncture
12	Guo X	2019	China/Chinese	116	Control − auriculotherapy − moxibustion – auriculotherapy + moxibustion
13	He SY	2021	China/Chinese	80	Control − acupoint application
14	He J	2017	China/Chinese	64	Control − moxibustion
15	Huang YY	2020	China/Chinese	88	Control – auriculotherapy + acupoint application
16	Li HW	2019	China/Chinese	59	Control − acupoint application
17	Li WJ	2020	China/Chinese	80	Control − auriculotherapy
18	Lin J	2018	China/Chinese	60	Control − auriculotherapy
19	Liu GL	2016	China/Chinese	40	Medicine – auriculotherapy + acupuncture
20	Ma JH	2021	China/Chinese	70	Medicine − acupoint application
21	Peng XH	2016	China/Chinese	190	Medicine − acupuncture
22	Shen LF	2016	China/Chinese	100	Control − electroacupuncture
23	Shi Y	2020	China/Chinese	80	Medicine − acupuncture
24	Song JR	2015	China/Chinese	120	Medicine – acupuncture + moxibustion
25	Song YP	2014	China/Chinese	100	Control − auriculotherapy
26	Wang BN	2019	China/Chinese	80	Sham − acupuncture
27	Wei XL	2019	China/Chinese	148	Control − auriculotherapy
28	Wu H	2019	China/Chinese	80	Control − acupoint application
29	Xie JJ	2021	China/Chinese	84	Control − acupuncture
30	Yu XX	2018	China/Chinese	102	Control − acupoint application
31	Zhang LF	2022	China/Chinese	90	Control – auriculotherapy + acupuncture + acupressure
32	Zhang M	2022	China/Chinese	60	Auriculotherapy – auriculotherapy + moxibustion
33	Zhang XJ	2019	China/Chinese	248	Control – auriculotherapy + moxibustion
34	Zhang ZY	2021	China/Chinese	102	Control − auriculotherapy
35	Zhong QL	2021	China/Chinese	80	Control – auriculotherapy + acupoint application
36	Zhong QL	2022	China/Chinese	100	Control – auriculotherapy + acupoint application
37	Zong TT	2023	China/Chinese	70	Medicine − moxibustion

### 3.3 The risk of bias

**Figure 2:**
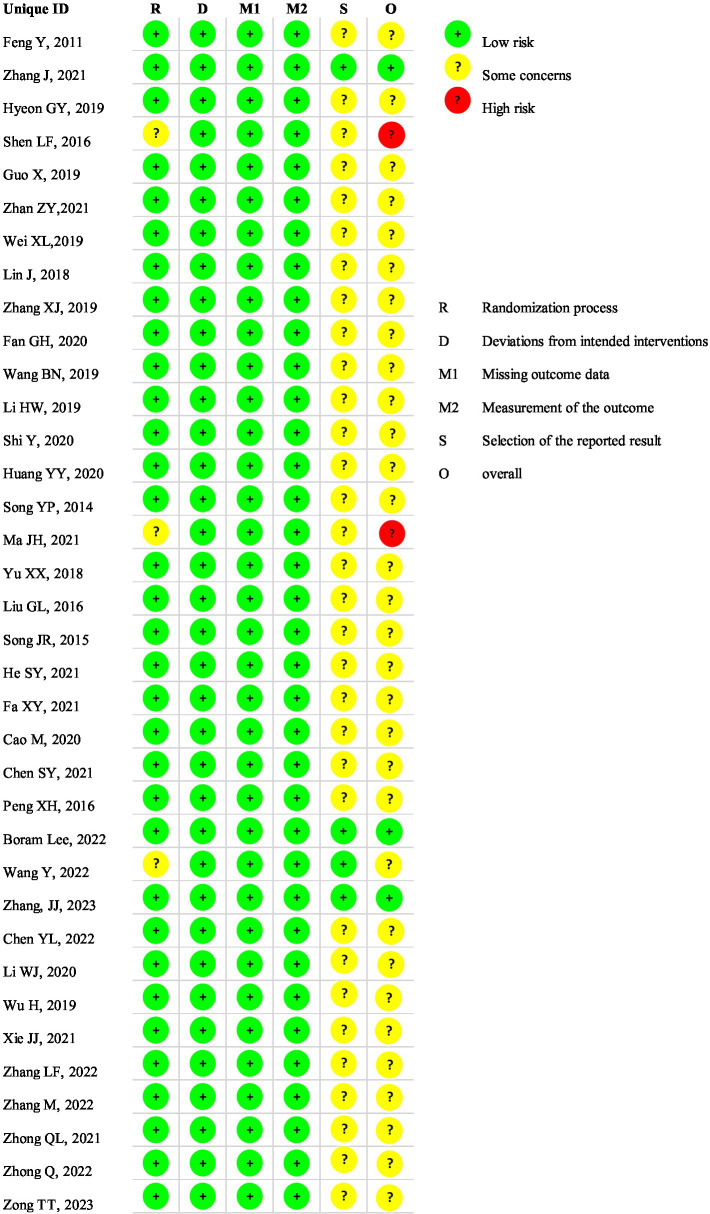
The risk of bias of included studies. The selection of the reported result constituted the primary source of bias.

As for the risk of bias, three studies were found to have a lower risk of bias while two studies had a higher risk of bias. The selection of the reported result constituted the primary source of bias. Figure 2 illustrated the risk of bias.

### 3.4 Network meta-analysis

#### 3.4.1 Primary outcome: PSQI

**Figure 3:**
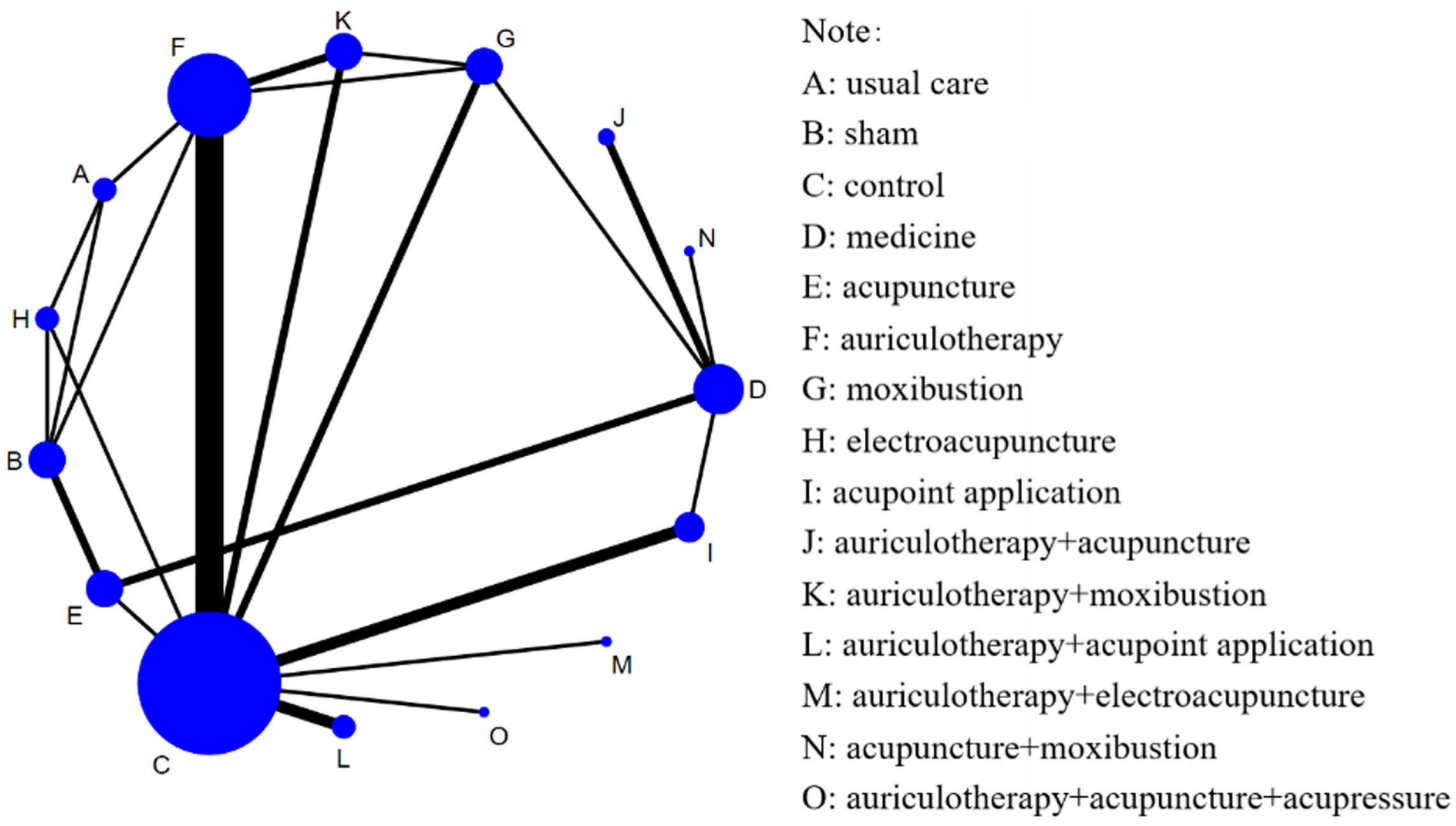
The network map of included studies. The size of each node was proportional to the number of participants receiving the respective intervention, while the boldness of the connecting lines corresponded to the number of comparative studies between the interventions. A total of 32 studies with 2,752 participants accepted one of 15 interventions, respectively, reporting the reduction of PSQI. The included studies generated 15 nodes, contributing to 23 pairs of comparisons.

Figure 3 exhibited the network map of eligible comparisons on PSQI. The network map indicated that majority of acupuncture therapies were compared with control or medications, yet a scarcity of comparisons existed between distinct acupuncture therapies.

**Figure 4:**
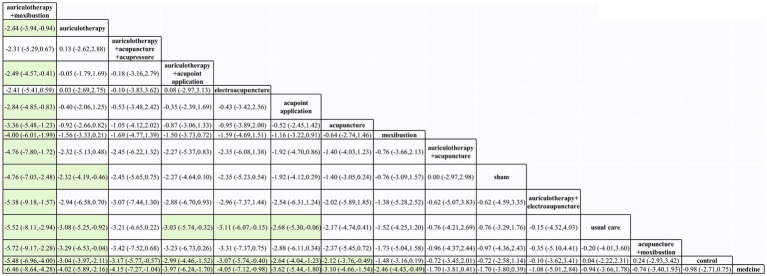
The league figure of included studies. Data with light green background meant significant difference between the comparisons. And compared with medicine, 8 interventions demonstrated significant results.

**Figure 5:**
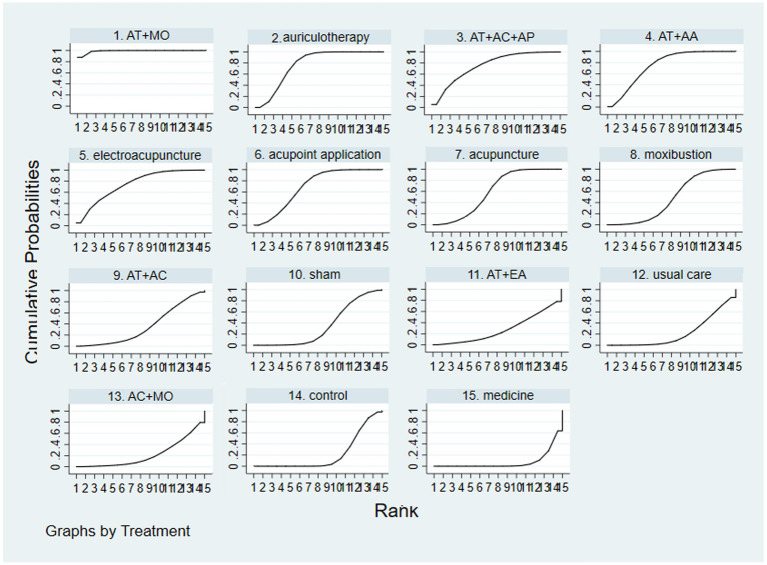
The SUCRA of included studies. Bigger SUCRA means higher rank, representing better efficacy of the intervention. The rank (SUCRA value) of each intervention in PSQI: auriculotherapy + moxibustion (98.98%) > auriculotherapy (77.47%) > auriculotherapy + acupuncture + acupressure (75.79%) > auriculotherapy + acupoint application (75.27%) > electroacupuncture (74.38%) > acupoint application (69.4%) > acupuncture (60.18%) > moxibustion (47.84%) > auriculotherapy + acupuncture (35.62%) > sham (34.31%) > auriculotherapy + electroacupuncture (27.65%) > usual care (22.35%) > acupuncture + moxibustion (21.57%) > control (21.52%) > medicine (7.67%). And auriculotherapy + moxibustion was the optimal acupuncture regime in reducing the score of PSQI according to SUCRA. AT, auriculotherapy; AC, acupuncture; MO, moxibustion; AP, acupressure; AA, acupoint application; EA, electroacupuncture.

League figure is derived from pairwise comparison of the various interventions. If both MD and the 95%CI are greater than or less than 0, the difference between the two interventions is deemed statistically significant. The league figure was presented in Figure 4. The SUCRA was calculated after comparing all interventions to rank their effectiveness of each intervention and determine the optimal one. The findings revealed that auriculotherapy + moxibustion exhibits the most significant impact on reducing the PSQI score in CRI patients. The SUCRA of PSQI was showed in Figure 5.

#### 3.4.2 Secondary outcome

The secondary outcome consisted of the subitems of PSQI, which contain subjective sleep quality, sleep latency, sleep duration, habitual sleep efficiency, sleep disturbances, daytime dysfunction and the use of sleep medications. Intervention of some studies included sleep medications, so the use of sleep medications was not statistically analyzed in this review.

Supplementary Figures S14A–F were the network map of each subitem of PSQI, with each subitem encompassing the same interventions. The results of subitems of PSQI were presented in Supplementary Figures S15A–F and Supplementary Figures S16A–F (Supplementary Figures S15A–F were league figure and Supplementary Figures S16A–F were SUCRA).

We found that the efficacy of auriculotherapy + acupuncture ranked highest in sleep latency, sleep duration, habitual sleep efficiency and daytime dysfunction, while the efficacy of auriculotherapy + moxibustion demonstrated the highest ranking in subjective sleep quality and sleep disturbances. The detailed rankings could be observed in Supplementary Figures S16A–F.

### 3.5 Network funnel plot of included studies

**Figure 6:**
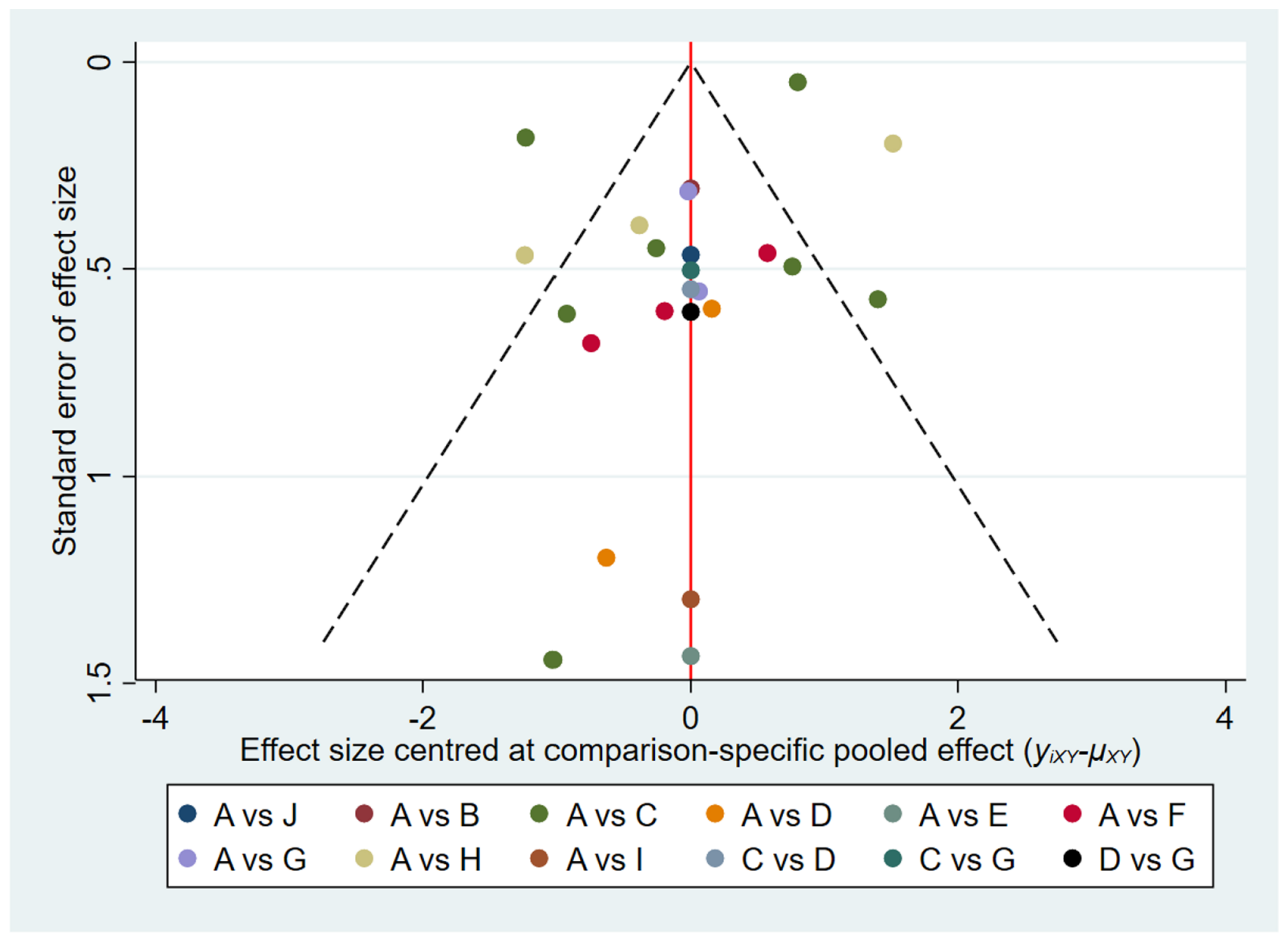
The adjusted funnel plot of included studies of PSQI. Though the distribution was nearly symmetric, some nodes were out of the funnel which showed some heterogeneity or publication bias in those studies. A, control; B, acupuncture; C, auriculotherapy; D, moxibustion; E, electroacupuncture; F, acupoint application; G, auriculotherapy + moxibustion; H, auriculotherapy + acupoint application; I, auriculotherapy + electroacupuncture; J, auriculotherapy + acupuncture + acupressure.

The adjusted funnel plot for PSQI was plotted and presented in Figure 6, revealing a general symmetry. However, some nodes were found to be outside the funnel, indicating the presence of heterogeneity or publication bias in those trials.

## 4 Discussion

In the wake of the high prevalence and the detrimental effect of CRI, there is a growing desire to find all prospective acupuncture therapies. In this review, we meticulously examined 37 randomized controlled trials involving acupuncture therapy for CRI and observed that auriculotherapy + moxibustion and auriculotherapy + acupuncture might be the optimal regimes for CRI.

Compared with previous meta-analyses (19, 20, 24–26), this study carried out a more comprehensive retrieval, including more recently published trials, successfully updating the evidence. The previous meta-analysis encompassed some studies in which participants were not diagnosed with CRI, PSQI served merely as a secondary outcome, and the experimental groups included other non-pharmaceutical interventions. In this review, however, the participants were confined to cancer survivors diagnosed with CRI, PSQI was the primary outcome, and we excluded studies that incorporated other non-pharmaceutical interventions. Furthermore, we came to different conclusions in comparison with previous network meta-analysis (21). The results of previous network meta-analysis displayed that transcutaneous electrical acupoint stimulation was the best acupuncture therapy for CRI. Several factors might account for the discrepancy: (1) Some of the included studies in the previous network meta-analysis pertained to tumor surgery, but this part of studies was excluded from this network meta-analysis, as it was challenging to differentiate between insomnia related to cancer or surgery, leading to a discrepancy in the included interventions. (2) The intervention in some of the included studies of previous network meta-analysis encompassed other non-pharmaceutical therapies, rendering the efficacy ranking of intervention was non-specific to acupuncture therapy. Consequently, these studies were also omitted in this review. Thus, the results of this review are more reliable to some extent.

Auriculotherapy + moxibustion was found to be the optimal acupuncture regime for reducing PSQI score, while auriculotherapy + acupuncture and auriculotherapy + moxibustion were the best acupuncture regimes for reducing the score of PSQI subitems. We conjectured that the auriculotherapy assumed a primary role, as its ranking surpassed that of acupuncture and moxibustion in the majority of outcomes.

The occurrence of insomnia is intimately linked to the hyperexcitation of the sympathetic and the hypo-excitability of the parasympathetic (27–29). Auriculotherapy is an intervention that involves stimulating auricular points with the acupuncture needle, thumb-tack needles, finger pressure, or beads for the therapeutic aim. Abundant nerve branches are distributed across the surface of the auricle (30). The concha auriculae area is primarily innervated by the auricular branch of the vagus nerve, while the triangular fossa area is innervated by the spinal nerve, vagus nerve and other nerves. According to Supplementary Table S9, the most commonly employed auricular points on CRI were Xin (heart, CO15) and Shenmen (TF4), locating in concha auriculae and triangular fossa respectively, which are both the distribution of the vagus nerve. As a vital component of the parasympathetic nervous system, the vagus nerve is instrumental in restoring balance between the sympathetic and parasympathetic nervous systems. Stimulation of the vagus nerve results in a reduced heart rate and blood pressure, contributing to a relaxed state, diminishing stress and anxiety, and thus alleviating insomnia symptoms, facilitating both the initiation and maintenance of sleep (31).

In a three-arm randomized trial involving 14 healthy males, researcher (32) performed acupuncture, electroacupuncture, and other stimuli on the auricular acupoints, corroborating the capability of auriculotherapy to stimulate the vagal activity.

At present, various imaging modalities, such as functional magnetic resonance imaging (fMRI), are employed to scrutinize the effect of auricular points. Study (33) reveals that transcutaneous auricular vagus nerve stimulation (taVNS) can regulate the activation state of the precuneus, a key encephalic region involved in episodic memory, emotional regulation, and introspection. The precuneus plays a role in regulating emotional, cognitive, and behavioral processes by suppressing excessive cortical activation, thereby aiding in the treatment of CRI. The etiology of CRI exhibits similarities with that of primary insomnia, all of which are predisposing factors, precipitating factors and perpetuating factors (34). Consequently, we postulated that auriculotherapy could be efficacious through a mechanism analogous to the aforementioned.

Employing PSQI subitems results, we discerned that various acupuncture modalities exhibit distinct curative effects on diverse CRI manifestations. Auriculotherapy + moxibustion demonstrated its merits in the improvement of subjective sleep quality and alleviating sleep disturbances (easy to wake up, difficulty in breathing, dreaminess, etc.), while auriculotherapy + acupuncture represented the optimal choice for ameliorating habitual sleep efficiency, abbreviating sleep latency, extending sleep duration and alleviating daytime dysfunction. Consequently, an appropriate acupuncture regimen can be tailored based on the findings of this review and the individual survivor’s symptoms. For instance, auriculotherapy + acupuncture could be selected for those experiencing difficulty in falling asleep, while auriculotherapy + moxibustion could be opted for those experiencing dreaminess or easy awakening. In addition, we found that the curative efficacy ranking of acupuncture therapy differed in the PSQI and its subitems. For example, auriculotherapy + acupuncture demonstrated a higher ranking in subitems but a lower ranking in the PSQI, which might be related to some studies listing only the total score or only some subitems of PSQI, which influenced the ranking of auriculotherapy + acupuncture.

### 4.1 Limitations

The results of this review provided more evidential recommendations for steering clinical practice. However, this review also encountered certain limitations. Studies other than those published in English and Chinese were excluded in this review, which could generate language bias. Furthermore, the insufficient quantity of studies, particularly in English, undermined the scope of the review. Methodologically, we assessed the risk of bias based on the Cochrane tool, and the risk of bias of most trials included was deemed uncertain. In addition, the morbidity of CRI varies across different cancers, while the cancer types were not explicitly stated in most studies, precluding the conduct of subgroup analysis. A majority of studies were conducted in China, with two in Korea. Acupuncture therapy’s deep historical and cultural roots in China, coupled with numerous practitioners and dedicated resources, likely contribute to the region’s higher volume of studies. Furthermore, trials from Europe and the United States that used non-pharmaceutical therapies like CBT as control group were excluded in this study. Consequently, the concentration of studies in China, as explained, may introduce a degree of publication bias.

## 5 Conclusion

We carried out an elaborate comparison and ultimately discovered that auriculotherapy + moxibustion constitutes the most efficacious acupuncture regimen for CRI patients with more substantial reductions in the score of PSQI, subjective sleep quality and sleep disturbances. In the meantime, auriculotherapy + acupuncture was found to be the most beneficial acupuncture regimen for CRI patients with more notable diminution in the score of sleep latency, sleep duration, habitual sleep efficiency and daytime dysfunction. Our results can assist healthcare professionals in choosing the optimal acupuncture regime for CRI.

## Data availability statement

The original contributions presented in the study are included in the article/Supplementary material, further inquiries can be directed to the corresponding authors.

## Author contributions

LC: Data curation, Formal analysis, Methodology, Project administration, Software, Writing – original draft. JL: Data curation, Formal analysis, Resources, Software, Writing – original draft. SX: Formal analysis, Investigation, Methodology, Writing – original draft. ZL: Investigation, Software, Writing – original draft. YJ: Project administration, Supervision, Writing – review & editing. ZZ: Project administration, Supervision, Writing – review & editing.

## References

[1] Palesh O Peppone L Innominato PF Janelsins M Jeong M Sprod L . Prevalence, putative mechanisms, and current management of sleep problems during chemotherapy for cancer. Nat Sci Sleep. (2012) 4:151–62. doi: 10.2147/NSS.S18895 23486503 PMC3593248

[2] Kreissl S Müller H Goergen H Meissner J Topp M Sökler M . Health-related quality of life in patients with Hodgkin lymphoma: a longitudinal analysis of the German Hodgkin study group. J Clin Oncol. (2020) 38:2839–48. doi: 10.1200/JCO.19.03160, PMID: 32574114

[3] Pachman DR Barton DL Swetz KM Loprinzi CL. Troublesome symptoms in cancer survivors: fatigue, insomnia, neuropathy, and pain. J Clin Oncol. (2012) 30:3687–96. doi: 10.1200/JCO.2012.41.7238 23008320

[4] Howell D Oliver TK Keller-Olaman S Davidson JR Garland S Samuels C . Sleep disturbance in adults with cancer: a systematic review of evidence for best practices in assessment and management for clinical practice. Ann Oncol. (2014) 25:791–800. doi: 10.1093/annonc/mdt506 24287882

[5] Mao JJ Pillai GG Andrade CJ Ligibel JA Basu P Cohen L . Integrative oncology: addressing the global challenges of cancer prevention and treatment. CA Cancer J Clin. (2021) 72:144–64. doi: 10.3322/caac.21706 34751943 PMC13183357

[6] Büttner-Teleagă A Kim YT Osel T Richter K. Sleep disorders in cancer—a systematic review. Int J Environ Res Public Health. (2021) 18:11696. doi: 10.3390/ijerph182111696, PMID: 34770209 PMC8583058

[7] Leysen L Lahousse A Nijs J Adriaenssens N Mairesse O Ivakhnov S . Prevalence and risk factors of sleep disturbances in breast cancersurvivors: systematic review and meta-analyses. Support Care Cancer. (2019) 27:4401–33. doi: 10.1007/s00520-019-04936-5 31346744

[8] Induru RR Walsh D. Cancer-related insomnia. Am J Hosp Palliat Care. (2014) 31:777–85. doi: 10.1177/1049909113508302 24142594

[9] Qaseem A Kansagara D Forciea MA Cooke M Denberg TD for the Clinical Guidelines Committee of the American College of Physicians. Management of chronic insomnia disorder in adults: a clinical practice guideline from the american college of physicians. Ann Intern Med. (2016) 165:125–33. doi: 10.7326/M15-2175, PMID: 27136449

[10] Riemann D Baglioni C Bassetti C Bjorvatn B Dolenc Groselj L Ellis JG . European guideline for the diagnosis and treatment of insomnia. J Sleep Res. (2017) 26:675–700. doi: 10.1111/jsr.12594 28875581

[11] Irwin M Olmstead R Carrillo C Sadeghi N Nicassio P Ganz PA . Tai chi Chih compared with cognitive behavioral therapy for the treatment of insomnia in survivors of breast Cancer- a randomized, partially blinded, noninferiority trial. J Clin Oncol. (2017) 35:2656–65. doi: 10.1200/JCO.2016.71.0285 28489508 PMC5549450

[12] Roscoe JA Garland SN Heckler CE Perlis ML Peoples AR Shayne M . Randomized placebo-controlled trial of cognitive behavioral therapy and Armodafinil for insomnia after Cancer treatment. J Clin Oncol. (2015) 33:165–71. doi: 10.1200/JCO.2014.57.6769 25452447 PMC4279236

[13] Koffel E Bramoweth AD Ulmer CS. Increasing access to and utilization of cognitive behavioral therapy for insomnia (CBT-I): a narrative review. J Gen Intern Med. (2018) 33:955–62. doi: 10.1007/s11606-018-4390-1 29619651 PMC5975165

[14] Höxtermann MD Buner K Haller H Kohl W Dobos G Reinisch M . Efficacy and safety of auricular acupuncture for the treatment of insomnia in breast cancer survivors: a randomized controlled trial. Cancers. (2021) 13:4082. doi: 10.3390/cancers13164082, PMID: 34439234 PMC8394534

[15] Liou KT Root JC Garland SN Green J Li Y Li QS . Effects of acupuncture versus cognitive behavioral therapy on cognitive function in cancer survivors with insomnia: a secondary analysis of a randomized clinical trial. Cancer. (2020) 126:3042–52. doi: 10.1002/cncr.32847, PMID: 32320061 PMC7992052

[16] Zhang J Qin Z So TH Chen H Lam WL Yam LL . Electroacupuncture plus auricular acupressure for chemotherapy-associated insomnia in breast Cancer patients: a pilot randomized controlled trial. Integr Cancer Ther. (2021) 20:153473542110191. doi: 10.1177/15347354211019103 PMC8161840 34036813

[17] Liu C Zhao Y Qin S Wang X Jiang Y Wu W. Randomized controlled trial of acupuncture for anxiety and depression in patients with chronic insomnia. Ann Transl Med. (2021) 9:1426. doi: 10.21037/atm-21-3845, PMID: 34733978 PMC8506741

[18] Yin X Li W Liang T Lu B Yue H Li S . Effect of Electroacupuncture on insomnia in patients with depression: a randomized clinical trial. JAMA Netw Open. (2022) 5:e2220563. doi: 10.1001/jamanetworkopen.2022.20563, PMID: 35797047 PMC9264041

[19] Choi TY Kim JI Lim HJ Lee MS. Acupuncture for managing Cancer-related insomnia: a systematic review of randomized clinical trials. Integr Cancer Ther. (2017) 16:135–46. doi: 10.1177/1534735416664172, PMID: 27531549 PMC5739128

[20] Lu HB Ma RC Yin YY Song CY Yang TT Xie J. Auricular acupressure for improving sleep quality in patients with lung cancer: a systematic review and meta-analysis. Holist Nurs Pract. (2022) 36:E27–e37. doi: 10.1097/HNP.0000000000000532 35708563

[21] Ou Y Lin D Ni X Li S Wu K Yuan L . Acupuncture and moxibustion in patients with cancer-related insomnia: a systematic review and network meta-analysis. Front Psych. (2023) 14:1108686. doi: 10.3389/fpsyt.2023.1108686, PMID: 36873228 PMC9979218

[22] Chen L Xu S Jia Z Tan Y Shi X Lin X. Comparative efficacy of different acupuncture therapies on cancer-related insomnia: protocol for a systematic review and network meta-analysis. BMJ Open. (2022) 12:e064181. doi: 10.1136/bmjopen-2022-064181, PMID: 36600339 PMC9743408

[23] Sterne JAC Savovic J Page MJ . RoB 2: a revised tool for assessing risk of bias in randomised trials. BMJ. (2019) 366:14898. doi: 10.1136/bmj.l4898 31462531

[24] Liu XL Cheng HL Moss S Wang CC Turner C Tan JY. Somatic acupoint stimulation for cancer-related sleep disturbance: a systematic review of randomized controlled trials. Evid Based Complement Alternat Med. (2020) 2020:1–12. doi: 10.1155/2020/2591320 PMC7206868 32419795

[25] Wang CC Han EY Jenkins M Hong X Pang S Whitehead L . The safety and efficacy of using moxibustion and or acupuncture for cancer-related insomnia: a systematic review and meta-analysis of randomised controlled trials. Palliat Care Soc Pract. (2022) 16:263235242110705. doi: 10.1177/26323524211070569 PMC8755931 35036916

[26] Zhang J Zhang Z Huang S Qiu X Lao L Huang Y . Acupuncture for cancer-related insomnia: a systematic review and meta-analysis. Phytomedicine. (2022) 102:154160. doi: 10.1016/j.phymed.2022.154160 35636168

[27] Chien L-W Chen F-C Hu H-Y Liu CF. Correlation of electrical conductance in Meridian and autonomic nervous activity after auricular acupressure in middle-aged women. J Altern Complement Med. (2014) 20:635–41. doi: 10.1089/acm.2012.0900, PMID: 24865945

[28] Kung Y-Y Yang CCH Chiu J-H Kuo TBJ. The relationship of subjective sleep quality and cardiac autonomic nervous system in postmenopausal women with insomnia under auricular acupressure. Menopause. (2011) 18:638–45. doi: 10.1097/gme.0b013e31820159c1, PMID: 21326120

[29] Seravalle G Mancia G Grassi G. Sympathetic nervous system, sleep, and hypertension. Curr Hypertens Rep. (2018) 20:74. doi: 10.1007/s11906-018-0874-y 29980938

[30] He W Wang X Shi H Shang H Li L Jing X . Auricular acupuncture and vagal regulation. Evid Based Complement Alternat Med. (2012) 2012:1–6. doi: 10.1155/2012/786839 PMC3523683 23304215

[31] Zhao Y-N Xiao X Li S-Y Zhang S Zhang JL He JK . Transcutaneous electrical cranial-auricular acupoints stimulation (TECAS) for treatment of the depressive disorder with insomnia as the complaint (DDI): a case series. Brain Stimul. (2022) 15:485–7. doi: 10.1016/j.brs.2022.01.018, PMID: 35167985

[32] La marca R Nedeljkovic M Yuan L Maercker A Elhert U. Effects of auricular electrical stimulation on vagal activity in healthy men: evidence from a three-armed randomized trial. Clin Sci. (2010) 118:537–46. doi: 10.1042/CS20090264 19895369

[33] Zhao B Bi Y Li L Zhang J Hong Y Zhang L . The instant spontaneous neuronal activity modulation of transcutaneous auricular Vagus nerve stimulation on patients with primary insomnia. Front Neurosci. (2020) 14:205. doi: 10.3389/fnins.2020.00205, PMID: 32231517 PMC7082749

[34] O’donnell J. Insomnia in cancer patients. Clin Cornerstone. (2004) 6:S6–S14. doi: 10.1016/S1098-3597(05)80002-X 15675652

