# Supplementary material for: Efficacy of acupuncture therapy on cancer-related insomnia: a systematic review and network meta-analysis
Source: Front Neurol. 2024 Feb 13;15:1342383. doi: 10.3389/fneur.2024.1342383 (PMC10896907; doi:10.3389/fneur.2024.1342383)
Supplement: Supplementary file 1 [file Data_Sheet_1.docx]

contents

[Part 1 The brief introduction of conventional acupuncture therapies. 3](#_Toc156155104)

[Figure S1: Acupuncture 3](#_Toc156155105)

[Figure S2: Auriculotherapy 4](#_Toc156155106)

[Figure S3: Moxibustion 5](#_Toc156155107)

[Figure S4: Electroacupuncture 6](#_Toc156155108)

[Figure S5: Acupressure 7](#_Toc156155109)

[Figure S6: Transcutaneous electric acupoint stimulation 8](#_Toc156155110)

[Part 2 Node split analysis of included studies 9](#_Toc156155111)

[Figure S7: node split analysis of included studies of PSQI. 9](#_Toc156155112)

[Figure S8: node split analysis of subjective sleep quality. 10](#_Toc156155113)

[Figure S9: node split analysis of sleep latency. 11](#_Toc156155114)

[Figure S11: node split analysis of habitual sleep efficiency. 13](#_Toc156155115)

[Figure S12: node split analysis of sleep disturbance. 14](#_Toc156155116)

[Figure S13: node split analysis of daytime dysfunction. 15](#_Toc156155117)

[Part 3 The network map of subitems of PSQI 16](#_Toc156155118)

[Figure S14: the network map of subitems of PSQI. 16](#_Toc156155119)

[Part 4 The league figure of included studies of subitems of PSQI 18](#_Toc156155120)

[Figure 15A: the league figure of included studies of subjective sleep quality. 18](#_Toc156155121)

[Figure 15B: the league figure of included studies of sleep latency. 18](#_Toc156155122)

[Figure 15C: the league figure of included studies of sleep duration. 18](#_Toc156155123)

[Figure 15D: the league figure of included studies of habitual sleep efficiency. 19](#_Toc156155124)

[Figure 15E: the league figure of included studies of sleep disturbance. 19](#_Toc156155125)

[Figure 15F: the league figure of included studies of daytime dysfunction. 20](#_Toc156155126)

[Part 5 The SUCRA of included studies of subitems 21](#_Toc156155127)

[Figure S16A-F: the SUCRA of included studies of subitems. 21](#_Toc156155128)

[Part 6 The search strategy of each database 24](#_Toc156155129)

[Table S1 the search strategy of PubMed 24](#_Toc156155130)

[Table S2 the search strategy of Cochrane 26](#_Toc156155131)

[Table S3 the search strategy of Embase 28](#_Toc156155132)

[Table S4 the search strategy of Web of Science 30](#_Toc156155133)

[Table S5 the search strategy of CBM 31](#_Toc156155134)

[Table S6 the search strategy of VIP 33](#_Toc156155135)

[Table S7 the search strategy of Wanfang Database 34](#_Toc156155136)

[Table S8 the search strategy of CNKI 35](#_Toc156155137)

[Part 7 The usage of acupoints in included studies 36](#_Toc156155138)

[Table S9 the usage of acupoints in included studies 36](#_Toc156155139)

# Part 1 The brief introduction of conventional acupuncture therapies.

**
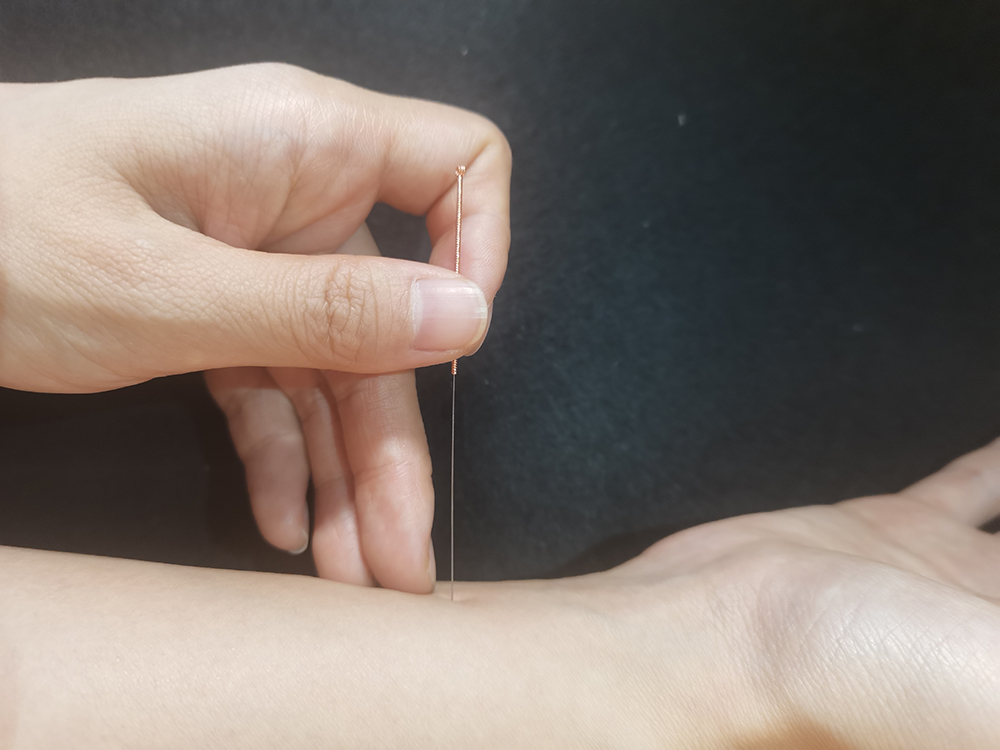
**

Figure S1: Acupuncture: insert a tailor-made needle into acupoint which is defined by Chinese Medicine. Then stimulate the acupoint manually.


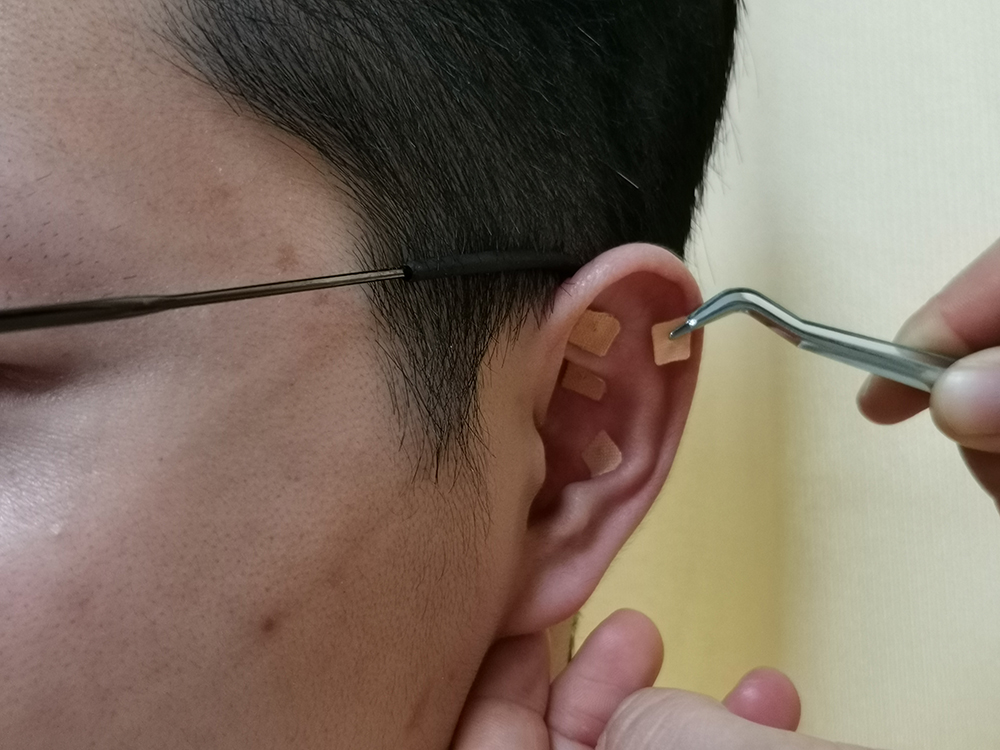


Figure S2: Auriculotherapy: adhere vaccaria seeds in place by tape or insert thumb-tack needle to stimulate auricular point on the auricle.


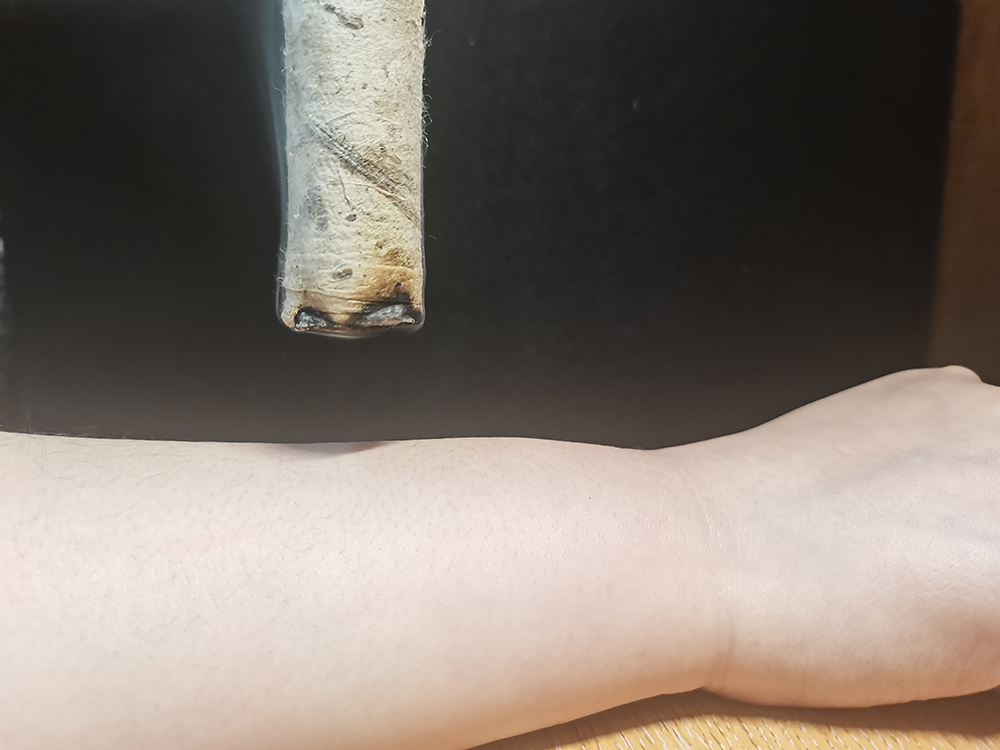


Figure S3: Moxibustion: place a moxa stick or moxa cone over acupoint to give the acupoint and meridian hot stimulus.


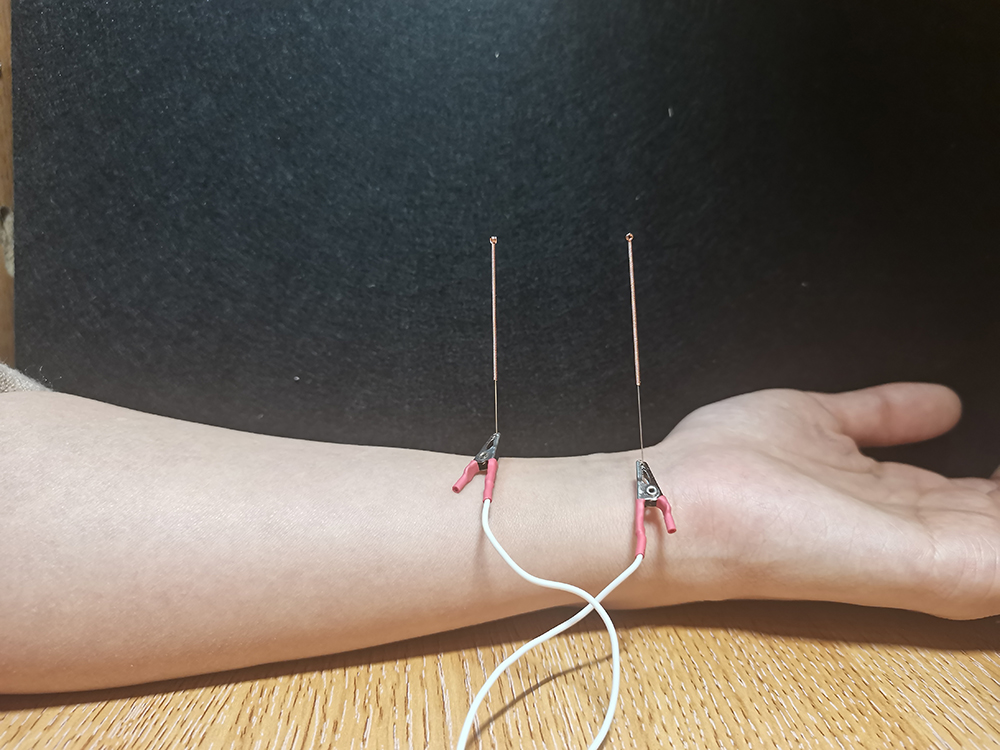


Figure S4: Electroacupuncture: insert a tailor-made needle into acupoint, and then stimulate acupoint rhythmically with electrical impulses passing through the needle.


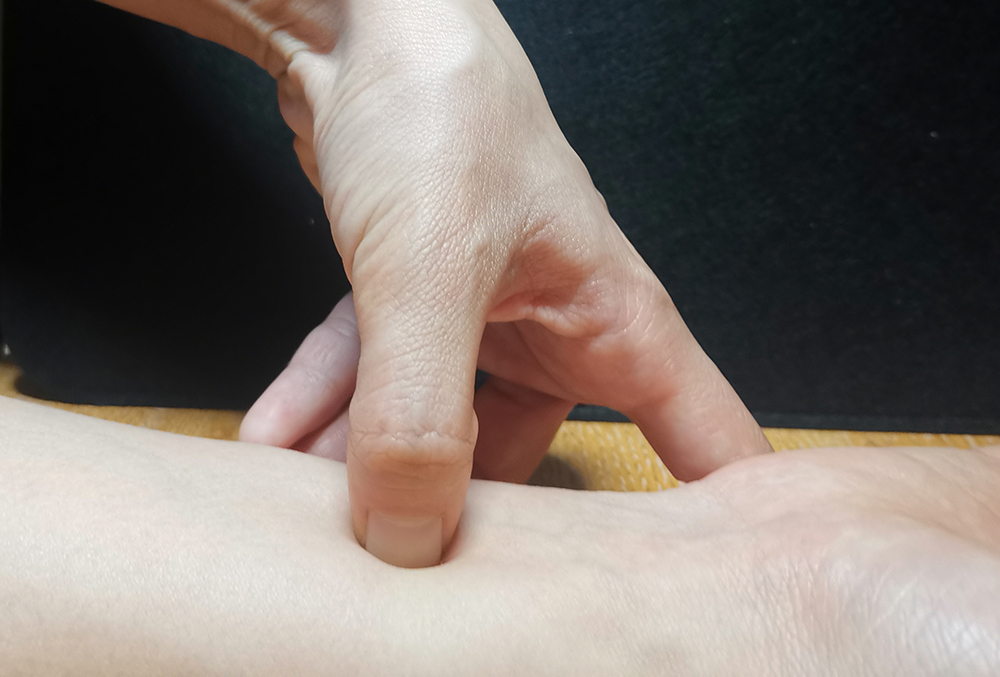


Figure S5: Acupressure: press acupoint with finger instead of needle or other acupuncture tool to stimulate acupoint.

**
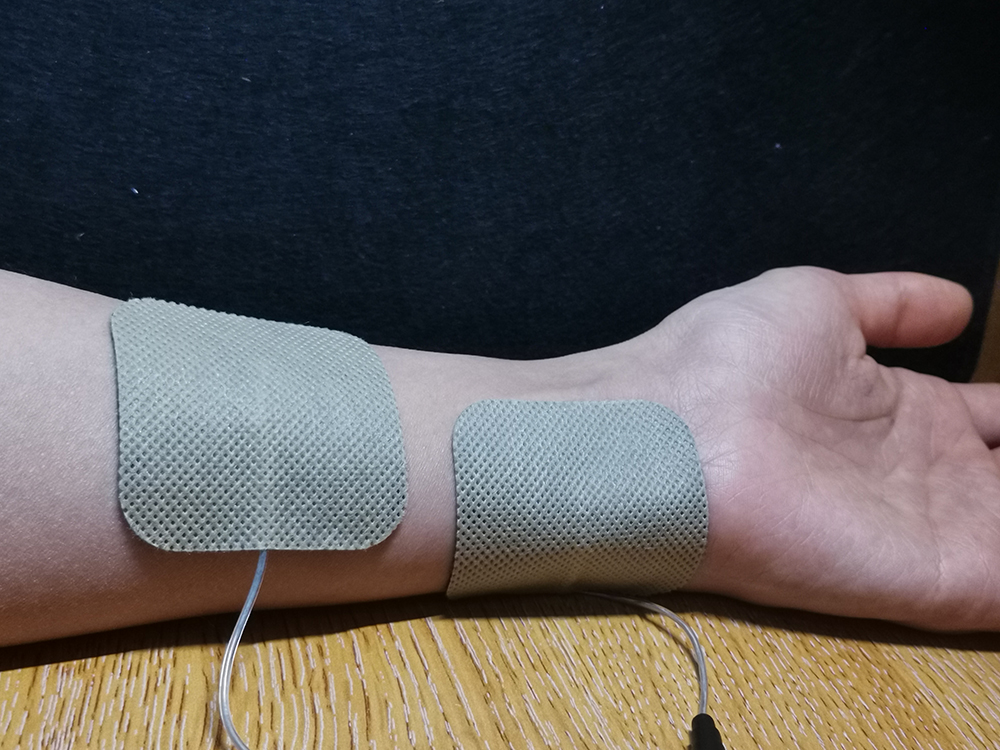
**

Figure S6: Transcutaneous electric acupoint stimulation: attach small electrodes on the skin of acupoint to deliver electrical impulses.

#
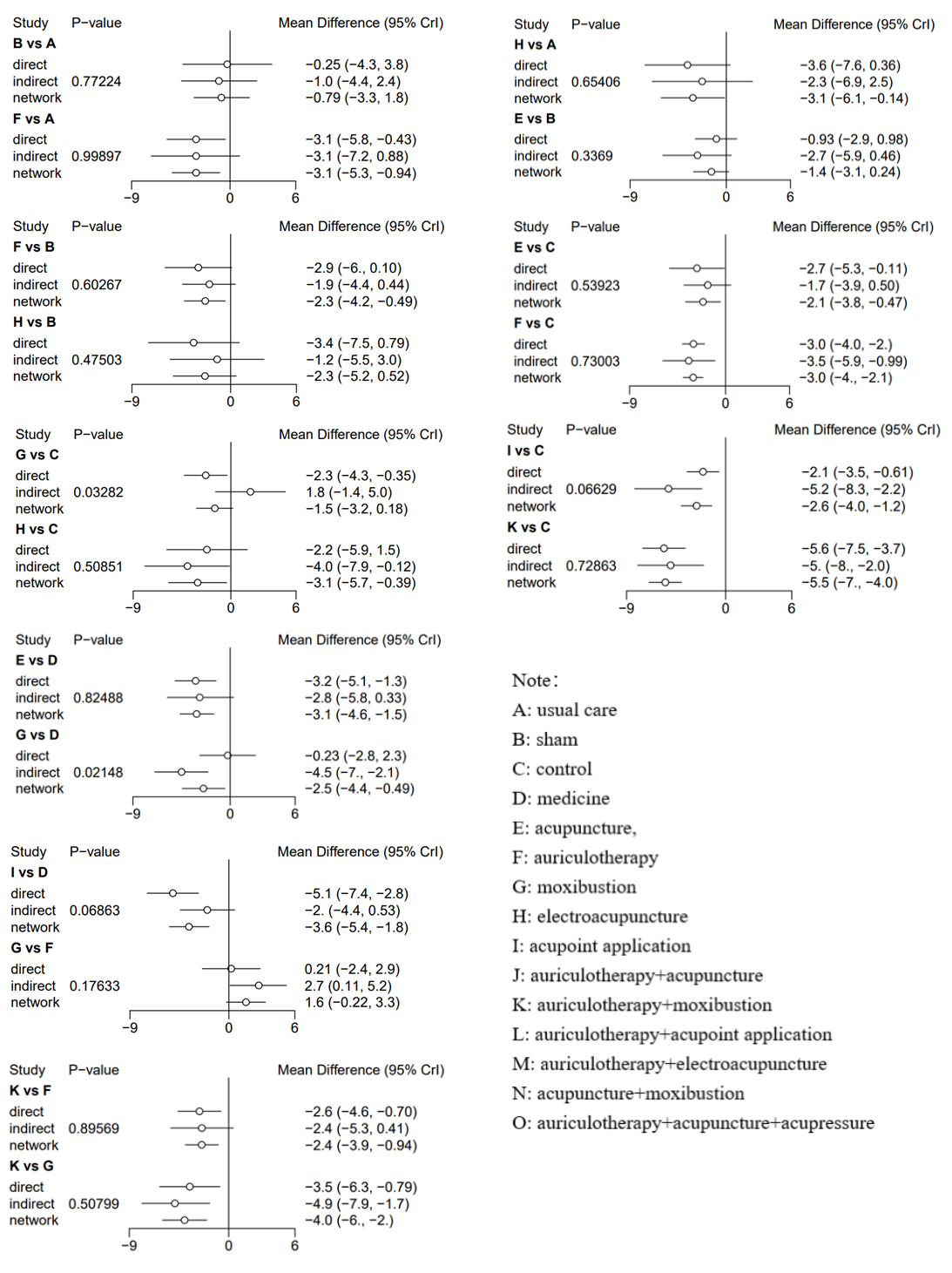
Part 2 Node split analysis of included studies

Figure S7: node split analysis of included studies of PSQI. The inconsistency of most comparisons was not significant difference (p>0.05). Thus, PSQI used to perform network meta-analysis.


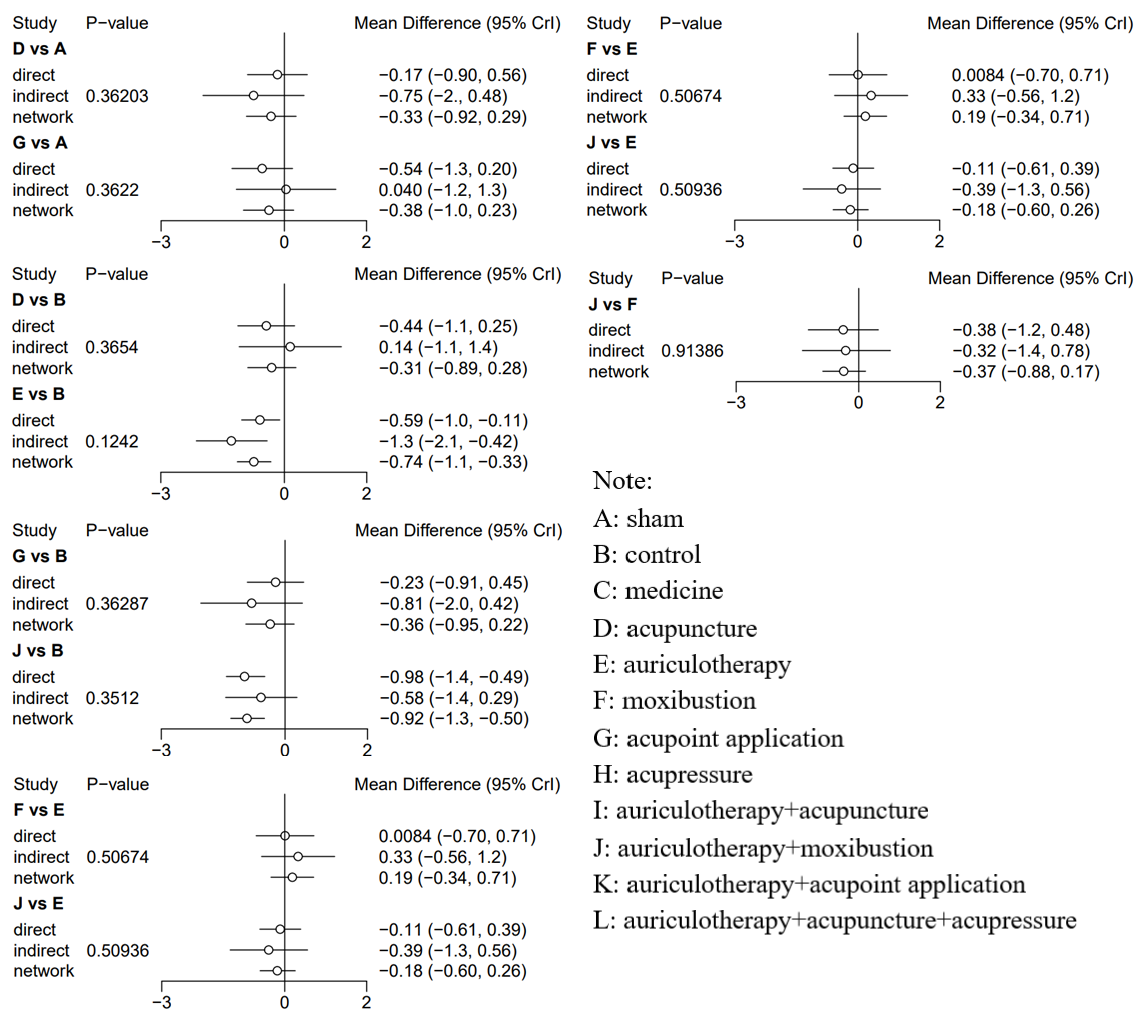


Figure S8: node split analysis of subjective sleep quality. The inconsistency of all comparisons was not significant difference (p>0.05). Thus, subjective sleep quality used to perform network meta-analysis.


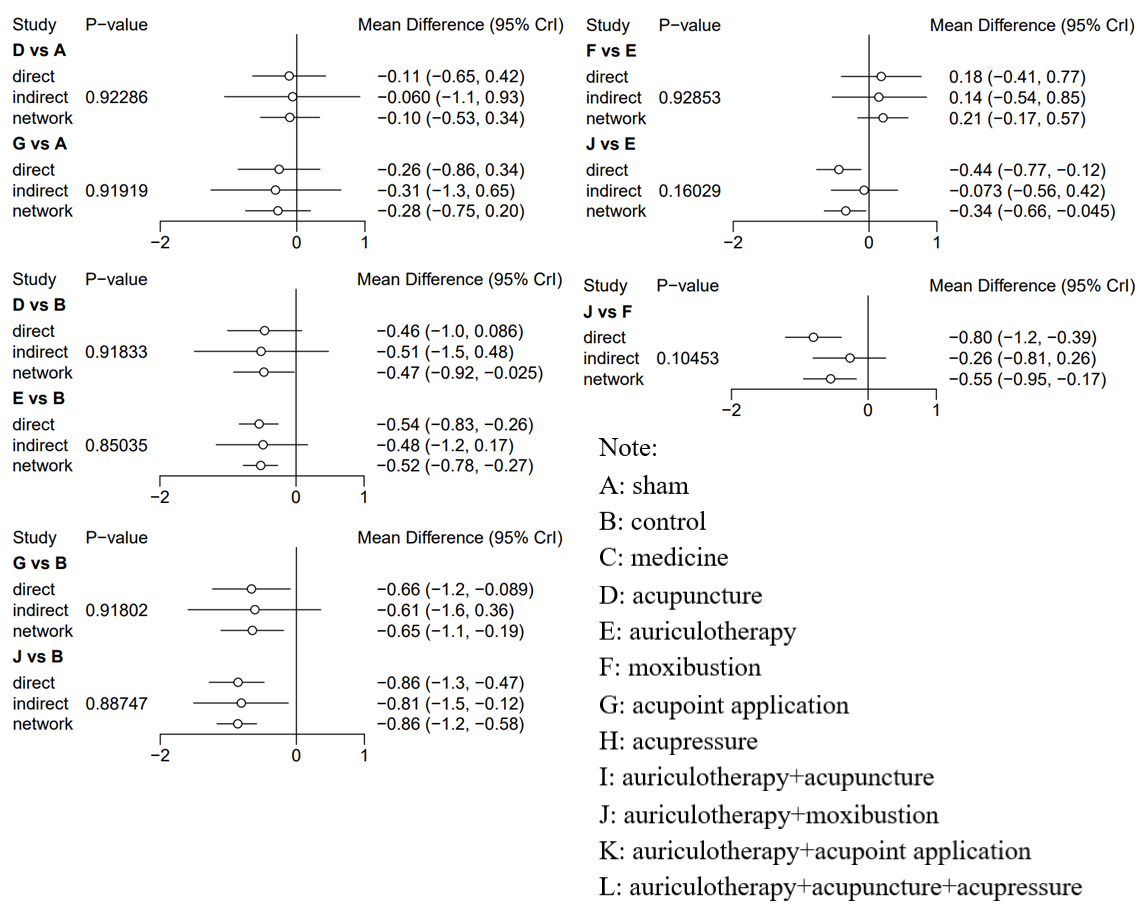


Figure S9: node split analysis of sleep latency. The inconsistency of all comparisons was not significant difference (p>0.05). Thus, sleep latency used to perform network meta-analysis.


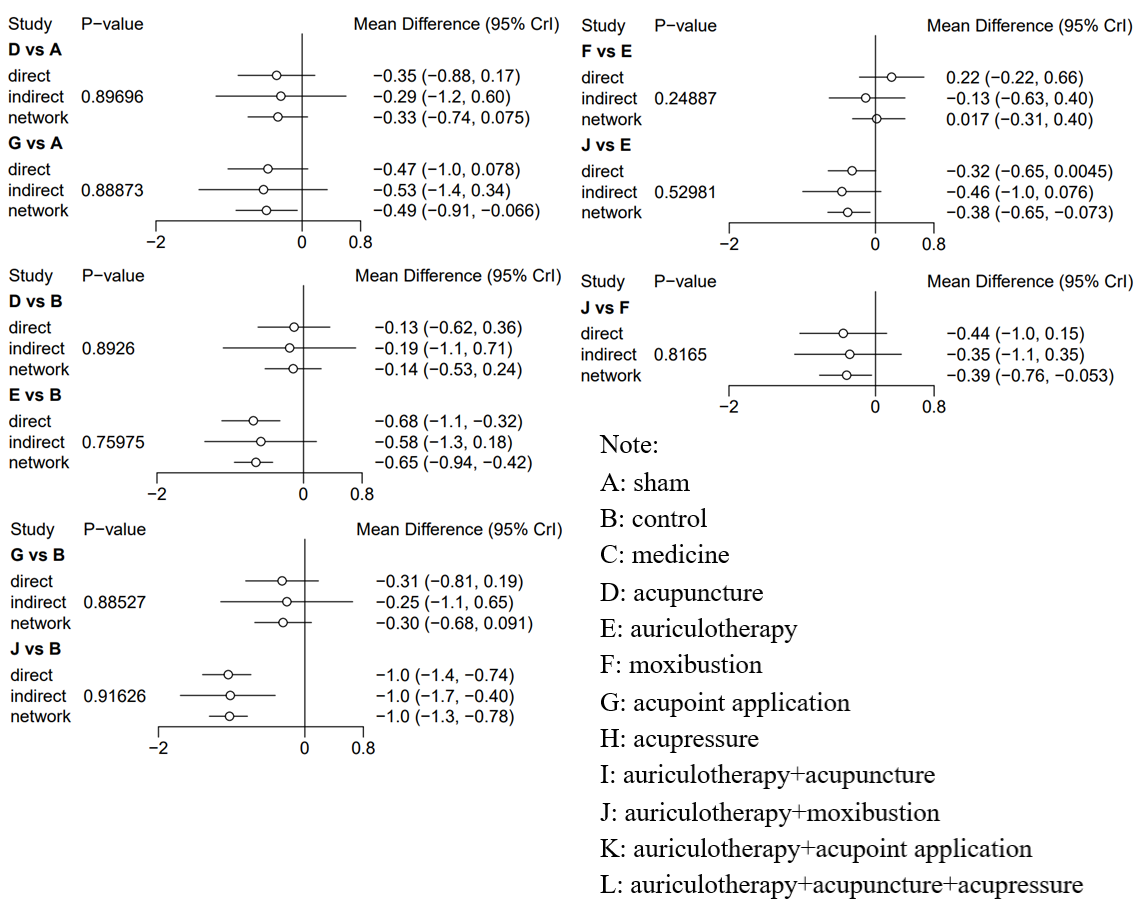
Figure S10: node split analysis of sleep duration. The inconsistency of all comparisons was not significant difference (p>0.05). Thus, sleep duration used to perform network meta-analysis.


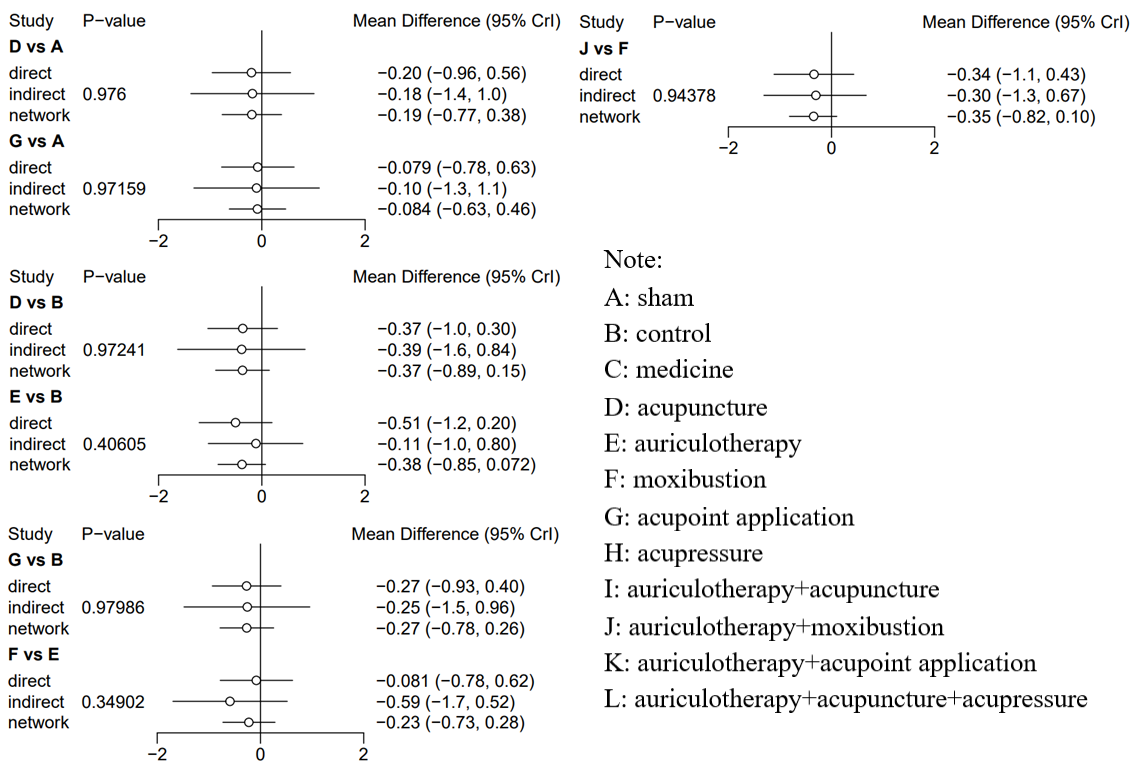


Figure S11: node split analysis of habitual sleep efficiency. The inconsistency of all comparisons was not significant difference (p>0.05). Thus, habitual sleep efficiency used to perform network meta-analysis.


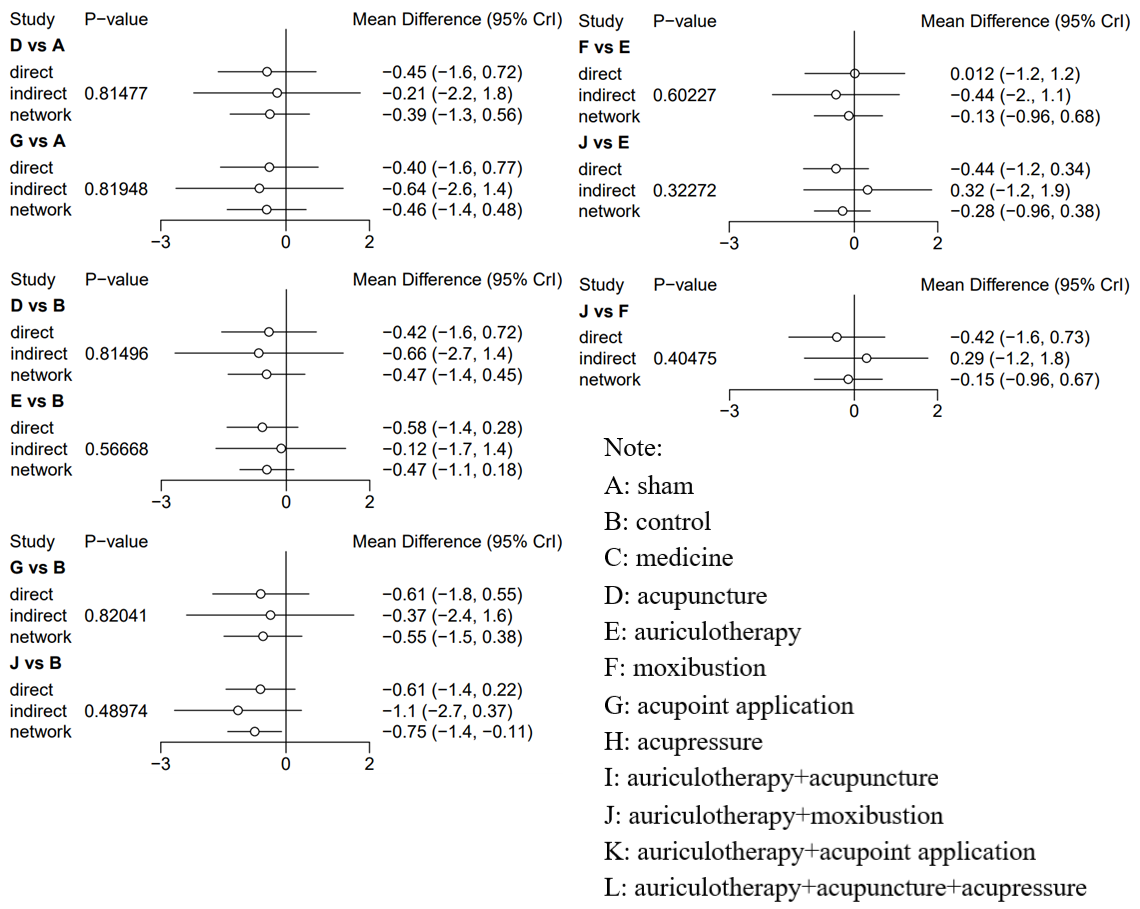


Figure S12: node split analysis of sleep disturbance. The inconsistency of all comparisons was not significant difference (p>0.05). Thus, sleep disturbance used to perform network meta-analysis.


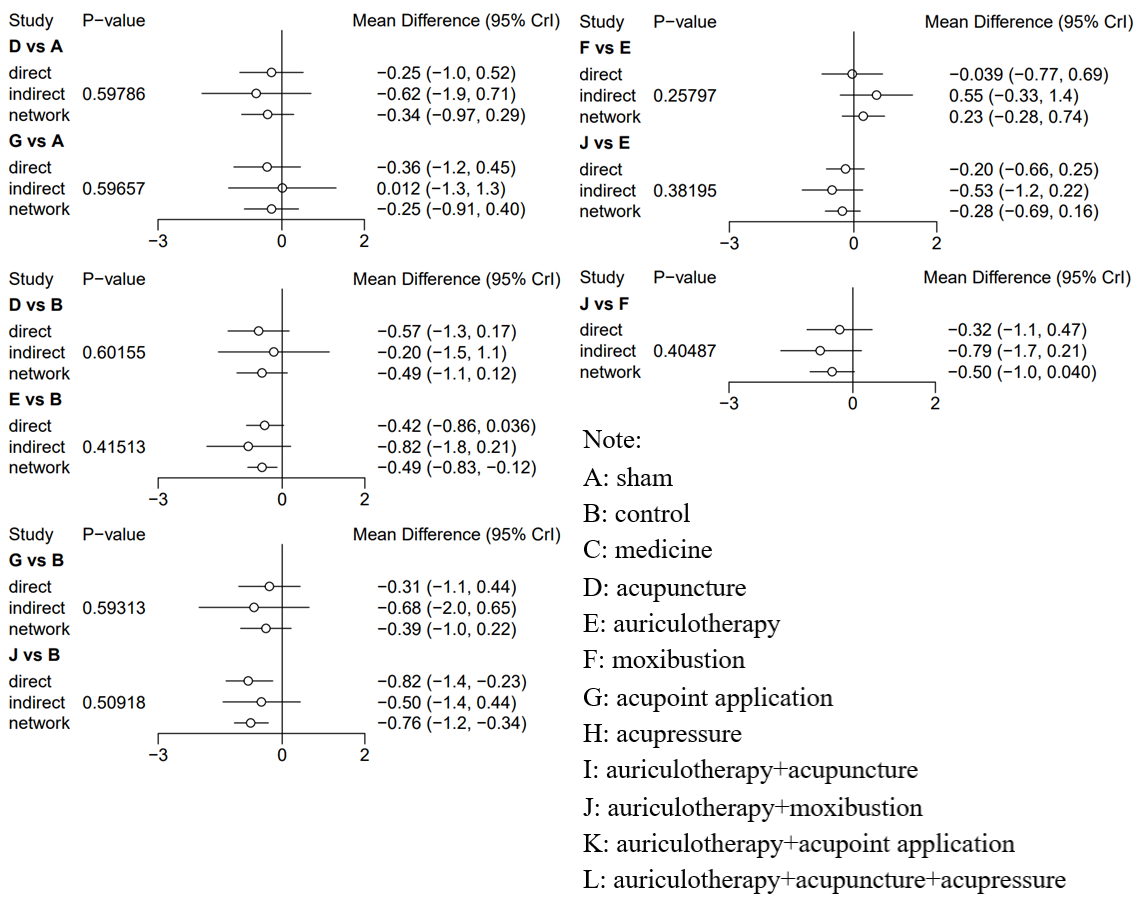


Figure S13: node split analysis of daytime dysfunction. The inconsistency of all comparisons was not significant difference (p>0.05). Thus, daytime dysfunction used to perform network meta-analysis.

# Part 3 The network map of subitems of PSQI


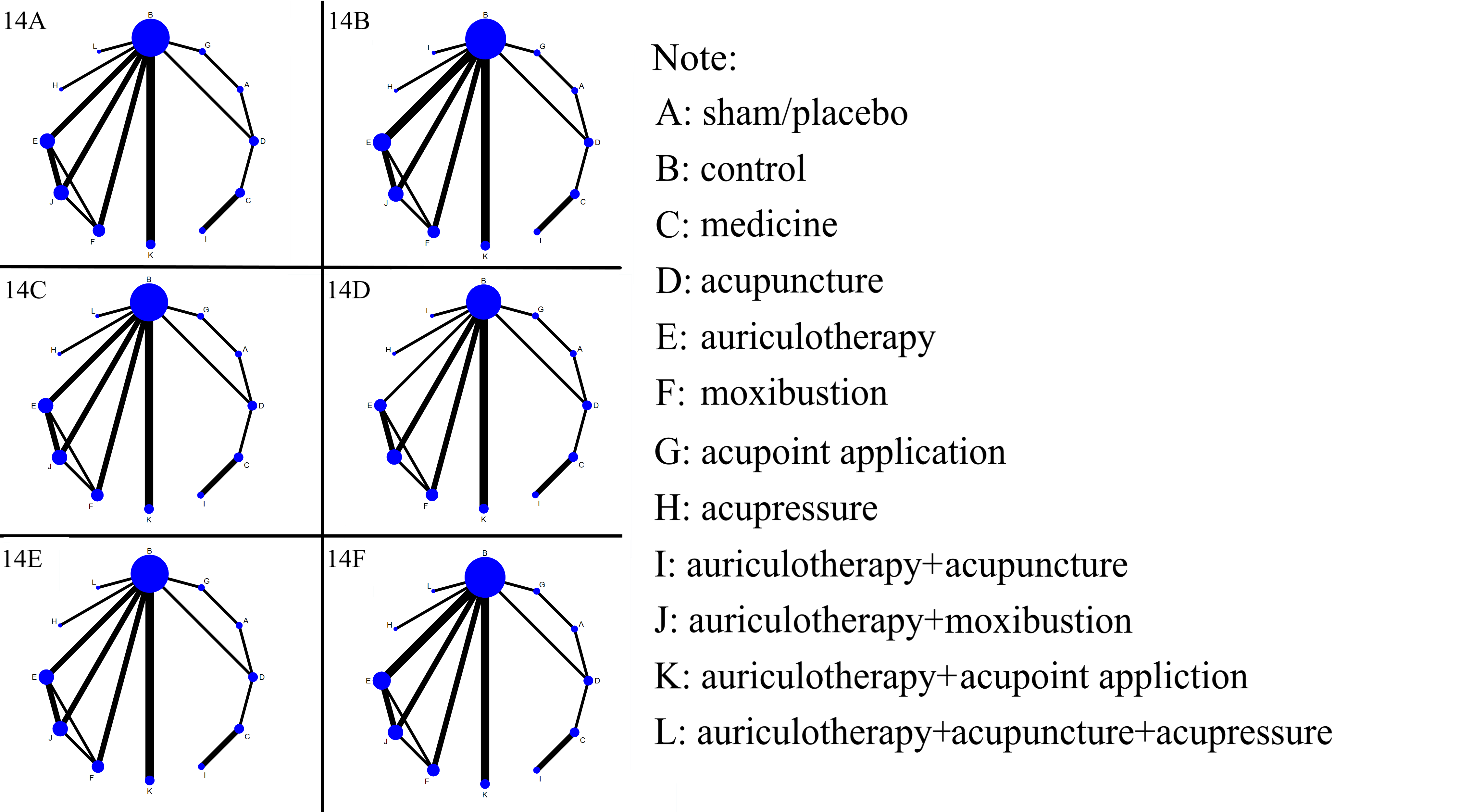


Figure S14: the network map of subitems of PSQI. The size of each node was proportional to the number of participants receiving the respective intervention, while the boldness of the connecting lines corresponded to the number of comparative studies between the interventions. Figure S14A: the network map of included studies of subjective sleep quality, and a total of 17 studies were included to compare subjective sleep quality score, involving 1771 participants and 12 interventions. Figure S14B: the network map of included studies of sleep latency, and a total of 18 studies were included to compare sleep latency score, involving 1871 participants and 12 interventions. Figure S14C: the network map of included studies of sleep duration, and a total of 17 studies were included to compare sleep duration score, involving 1723 participants and 12 interventions. Figure S14D: the network map of included studies of habitual sleep efficiency, and a total of 16 studies were included to compare habitual sleep efficiency score, involving 1623 participants and 12 interventions. Figure S14E: the network map of included studies of sleep disturbance, and a total of 17 studies were included to compare sleep disturbance score, involving 1723 participants and 12 interventions. Figure S14F: the network map of included studies of daytime dysfunction, and a total of 18 studies were included to compare daytime dysfunction score, involving 1871 participants and 12 interventions.

# Part 4 The league figure of included studies of subitems of PSQI

**
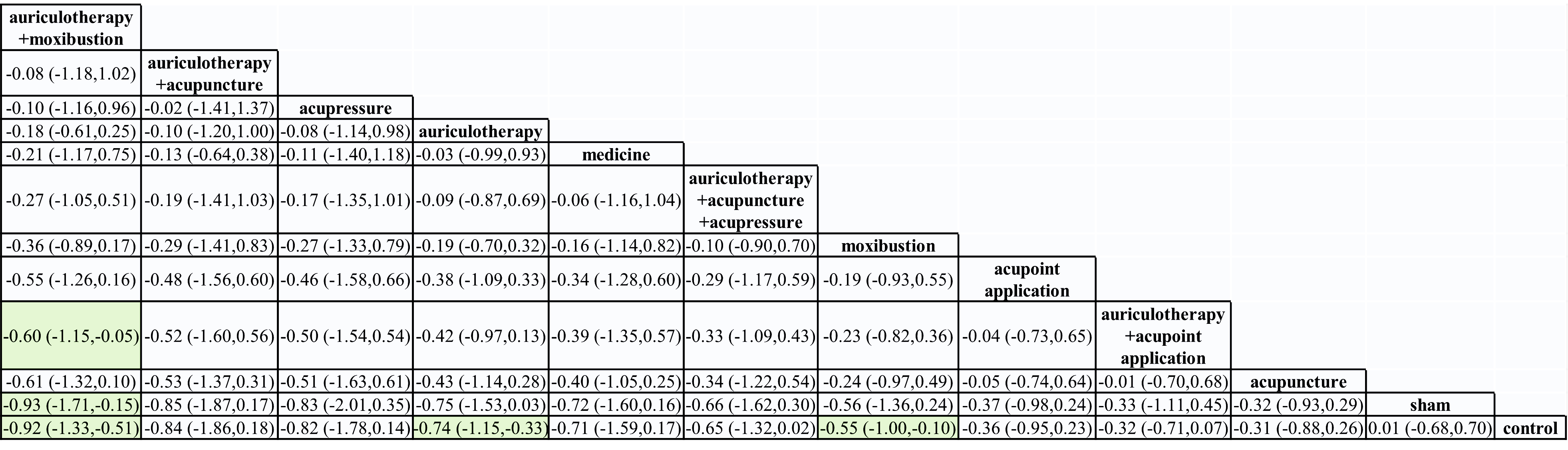
**

Figure 15A: the league figure of included studies of subjective sleep quality. Data with light green background meant significant difference between the comparisons. And compared with control, 3 interventions demonstrated significant results.


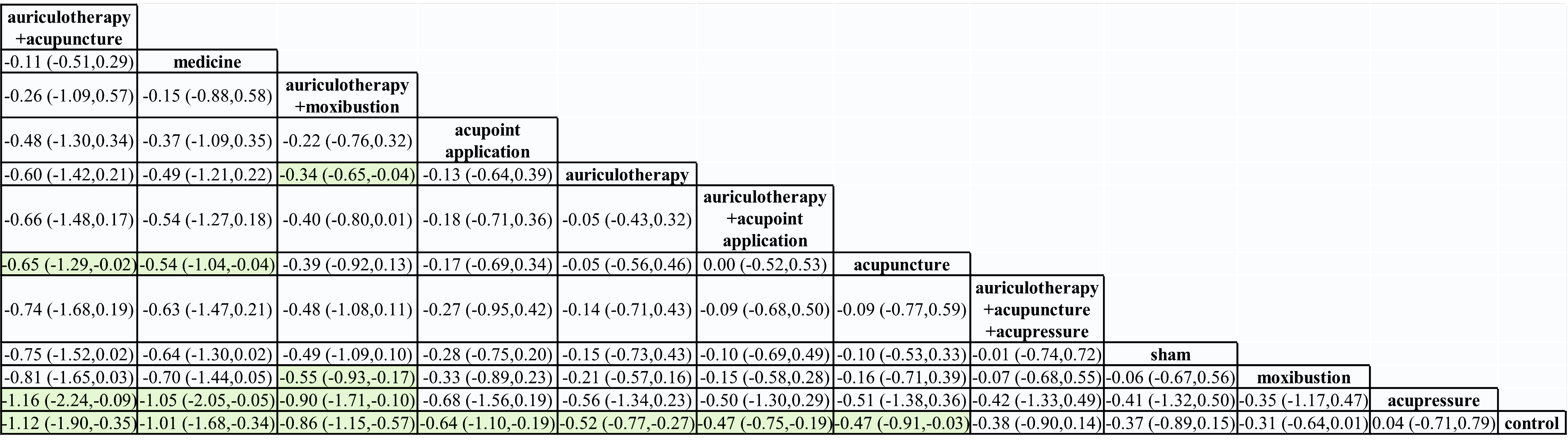


Figure 15B: the league figure of included studies of sleep latency. Data with light green background meant significant difference between the comparisons. And compared with control, 7 interventions demonstrated significant results.


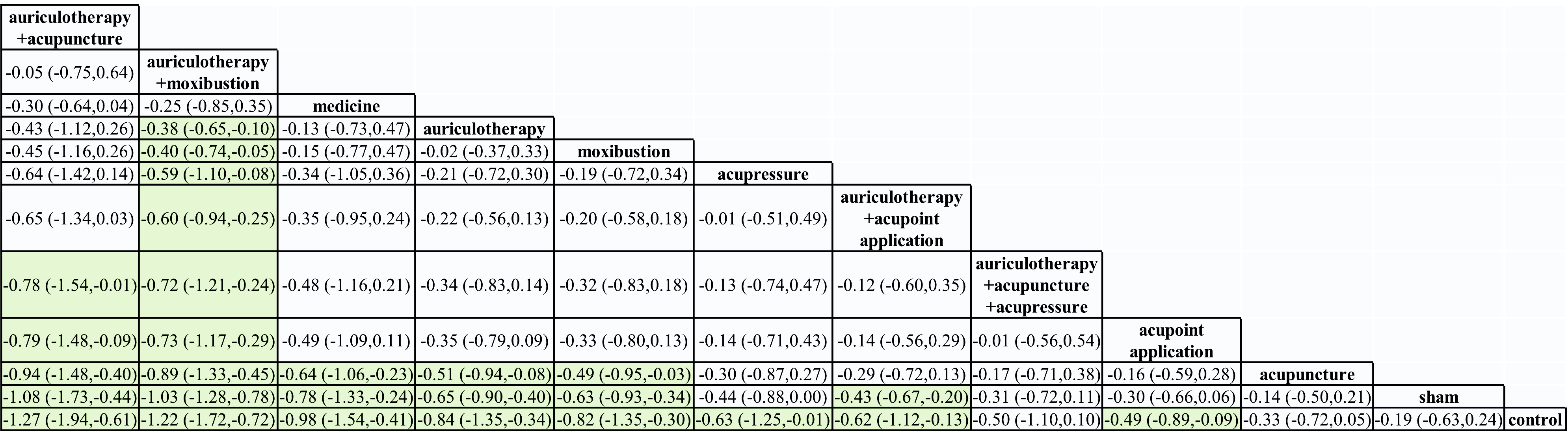


Figure 15C: the league figure of included studies of sleep duration. Data with light green background meant significant difference between the comparisons. And compared with control, 8 interventions demonstrated significant results.


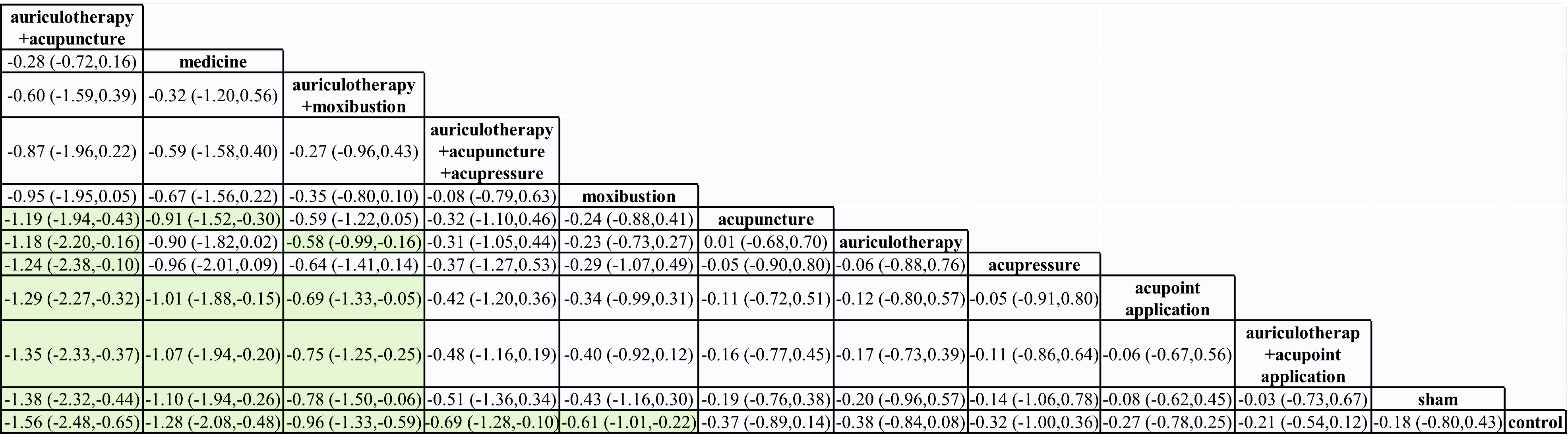


Figure 15D: the league figure of included studies of habitual sleep efficiency. Data with light green background meant significant difference between the comparisons. And compared with control, 5 interventions demonstrated significant results.


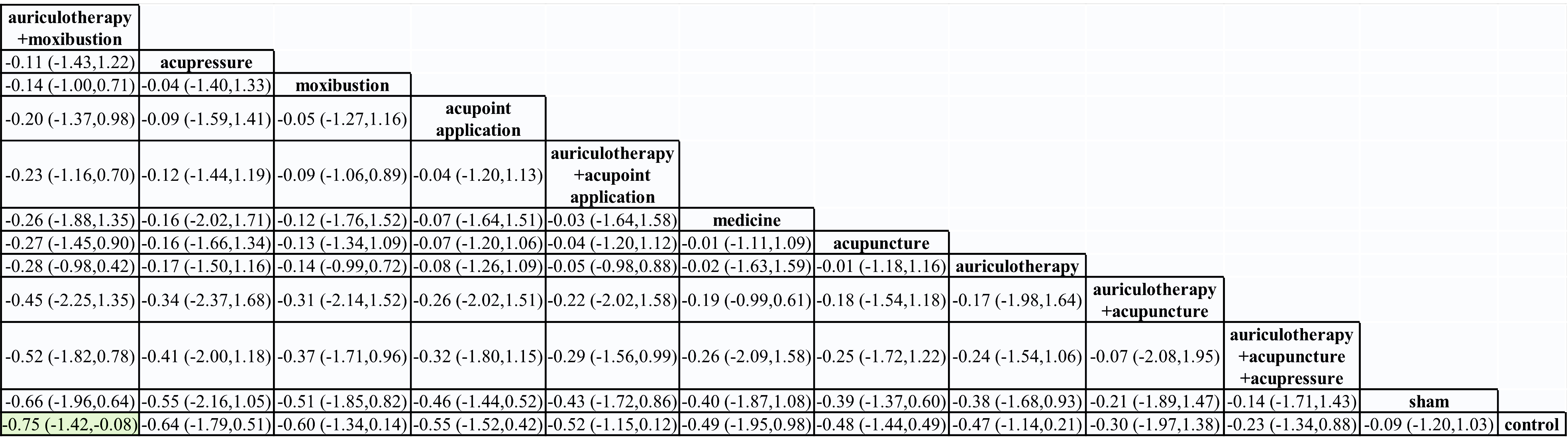


Figure 15E: the league figure of included studies of sleep disturbance. Data with light green background meant significant difference between the comparisons. And compared with control, only 1 intervention demonstrated significant results.


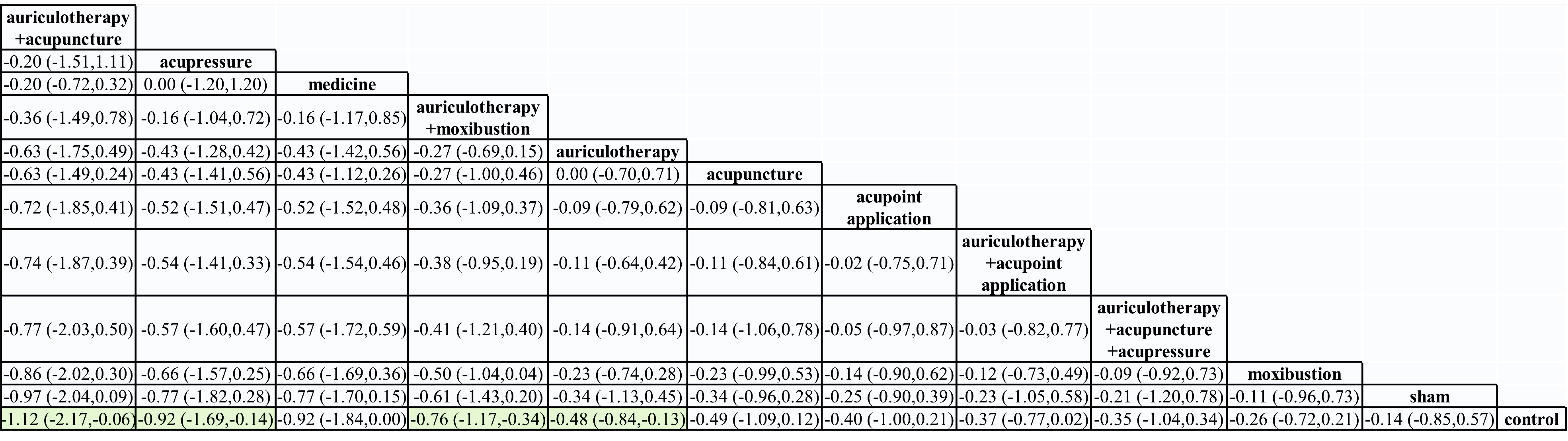


Figure 15F: the league figure of included studies of daytime dysfunction. Data with light green background meant significant difference between the comparisons. And compared with control, 3 interventions demonstrated significant results.

# Part 5 The SUCRA of included studies of subitems


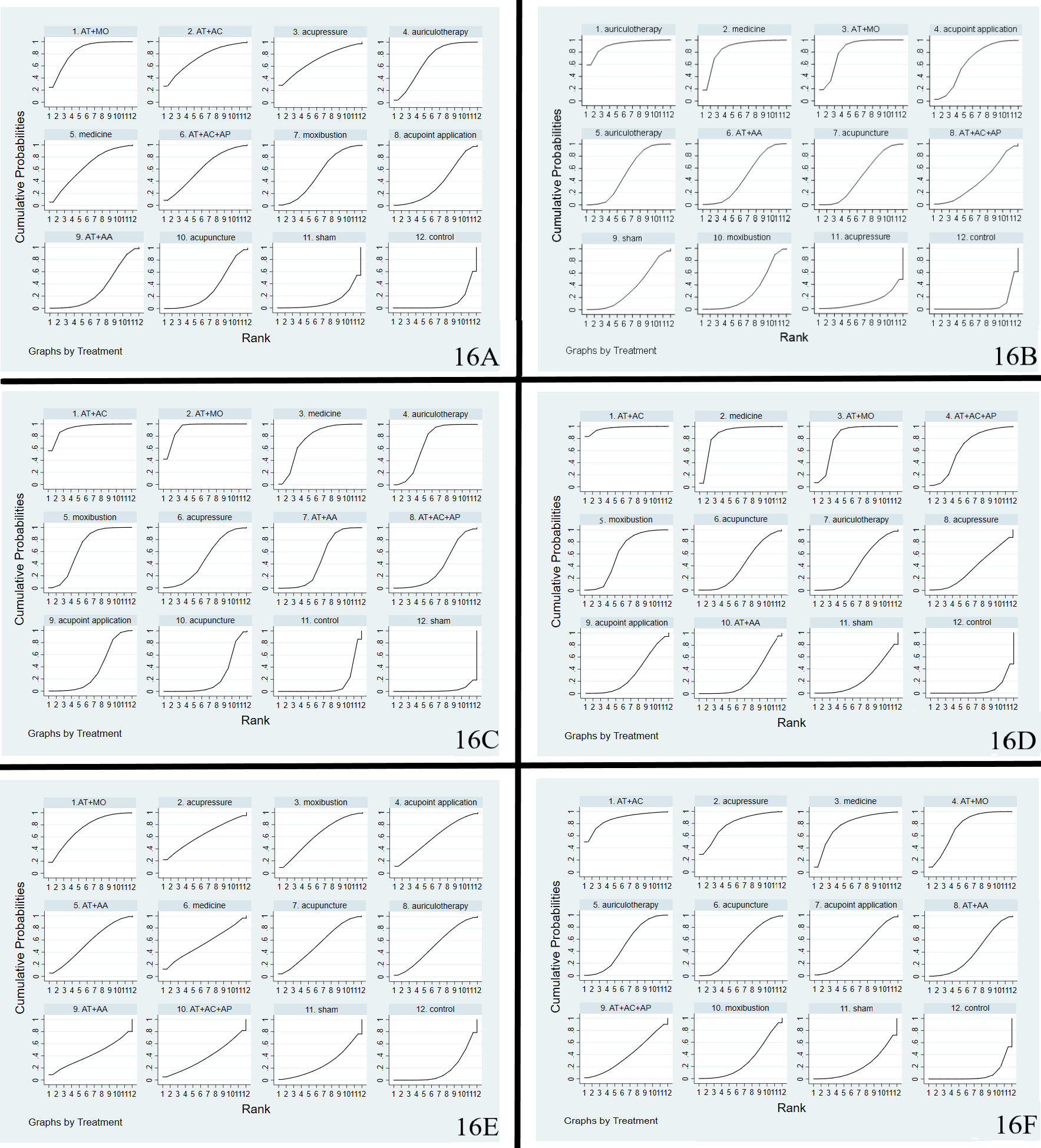


Figure S16A-F: the SUCRA of included studies of subitems. Bigger SUCRA means higher rank, representing better efficacy of the intervention. Figure S16A: the SUCRA of included studies of subjective sleep quality. The rank (SUCRA value) of each intervention in subjective sleep quality: auriculotherapy + moxibustion (83.86%) > auriculotherapy + acupuncture (73.56%) > acupressure (69.99%) > auriculotherapy (69.55%) > medicine (64.65%) > auriculotherapy + acupuncture + acupressure (61.02%) > moxibustion (53.03%) > acupoint application (38.19%) > auriculotherapy + acupoint application (33.31%) > acupuncture (32.73%) > sham (11.41%) > control (8.691%). Figure S16B: the SUCRA of included studies of sleep latency. The rank (SUCRA value) of each intervention in sleep latency: auriculotherapy + acupuncture (91.82%) > medicine (86.49%) > auriculotherapy + moxibustion (83.37%) > acupoint application (65.34%) > auriculotherapy (53.38%) > auriculotherapy + acupoint application (47.2%) > acupuncture (46.97%) > auriculotherapy + acupuncture + acupressure (38.99%) > sham (36.07%) > moxibustion (30.64%) > acupressure (12.98%) + control (6.741%). Figure S16C: the SUCRA of included studies of sleep duration. The rank (SUCRA value) of each intervention in sleep duration: auriculotherapy + acupuncture (93.09%) > auriculotherapy + moxibustion (92.87%) > medicine (75.2%) > auriculotherapy (68.66%) > moxibustion (66.74%) > acupressure (48.51%) > auriculotherapy + acupoint application (47.51%) > auriculotherapy + acupuncture + acupressure (36.33%) > acupoint application (35.5%) > acupuncture (22.38%) > control (10.47%) + sham (2.751%). Figure S16D: the SUCRA of included studies of habitual sleep efficiency. The rank (SUCRA value) of each intervention in habitual sleep efficiency: auriculotherapy + acupuncture (97.03%) > medicine (87.55%) > auriculotherapy + moxibustion (81.2%) > auriculotherapy + acupuncture + acupressure (64.71%) > moxibustion (60.81%) > acupuncture (41.37%) > auriculotherapy (41.33%) > acupressure (36.62%) > acupoint application (31.94%) > auriculotherapy + acupoint application (26.23%) > sham (24.4%) > control (6.816%).

Figure S16E: the SUCRA of included studies of sleep disturbance. The rank (SUCRA value) of each intervention in sleep disturbance: auriculotherapy + moxibustion (73.93%) > acupressure (62.77%) > auriculotherapy (62.59%) > acupoint application (59.12%) > auriculotherapy + acupoint application (56.51%) > medicine (54.69%) > acupuncture (54.15%) > auriculotherapy (51.96%) > auriculotherapy + acupuncture (42.06%) > auriculotherapy + acupuncture + acupressure (37.63%) > sham/placebo (27.61%) > control (16.99%). Figure S16F: the SUCRA of included studies of daytime dysfunction. The rank (SUCRA value) of each intervention in daytime dysfunction: auriculotherapy + acupuncture (87.25%) > acupressure (78.99%) > medicine (77.49%) > auriculotherapy + moxibustion (74.45%) > auriculotherapy (51.02%) > acupuncture (50.02%) > acupoint application (42.81%) > auriculotherapy + acupoint application (40.41%) > auriculotherapy + acupuncture + acupressure (39%) > moxibustion (29.54%) > sham (21.48%) > control (7.55%). AT: auriculotherapy, AC: acupuncture, MO: moxibustion, AP: acupressure, AA: acupoint application.

# Part 6 The search strategy of each database

## Table S1 the search strategy of PubMed

| sequence number | content  retrieval |
| --- | --- |
| #1 | neoplasms[MeSH Terms] |
| #2 | carcinoma[MeSH Terms] |
| #3 | sarcoma[MeSH Terms] |
| #4 | carcinosarcoma[MeSH Terms] |
| #5 | lymphoma[MeSH Terms] |
| #6 | "hodgkin disease" [MeSH Terms] |
| #7 | leukemia[MeSH Terms] |
| #8 | "Neoplasm Metastasis"[MeSH Terms] |
| #9 | #1 OR #2 OR #3 OR #4 OR #5 OR #6 OR #7 OR #8 |
| #10 | neoplasms[Title/Abstract] |
| #11 | carcinoma[Title/Abstract] |
| #12 | cancer[Title/Abstract] |
| #13 | tumor[Title/Abstract] |
| #14 | oncology[Title/Abstract] |
| #15 | lymphoma[Title/Abstract] |
| #16 | "Neoplasm Metastasis"[Title/Abstract] |
| #17 | malignancy[Title/Abstract] |
| #18 | neoplasia[Title/Abstract] |
| #19 | carcinomatoses[Title/Abstract] |
| #20 | carcinosarcoma[Title/Abstract] |
| #21 | leukemia[Title/Abstract] |
| #22 | "hodgkin's disease"[Title/Abstract] |
| #23 | #10 OR #11 OR #12 OR #13 OR #14 OR #15 OR #16 OR #17 OR #18 OR #19 OR #20 OR #21 OR #22 |
| #24 | #9 OR #23 |
| #25 | "sleep initiation and maintenance disorders"[MeSH Terms] |
| #26 | sleep[MeSH Terms] |
| #27 | dyssomnias[MeSH Terms] |
| #28 | "sleep disorders, intrinsic"[MeSH Terms] |
| #29 | "sleep disorders, circadian rhythm"[MeSH Terms] |
| #30 | sleep deprivation[MeSH Terms] |
| #31 | wakefulness[MeSH Terms] |
| #32 | "sleep-wake disorders"[MeSH Terms] |
| #33 | #25 OR #26 OR #27 OR #28 OR #29 OR #30 OR #31 OR #32 |
| #34 | insomnia[Title/Abstract] |
| #35 | "sleep initiation and maintenance disorders"[Title/Abstract] |
| #36 | sleep*[Title/Abstract] |
| #37 | "early awakening"[Title/Abstract] |
| #38 | dyssomnias[Title/Abstract] |
| #39 | #34 OR #35 OR #36 OR #37 OR #38 |
| #40 | #33 OR #39 |
| #41 | acupuncture[MeSH Terms] |
| #42 | "acupuncture therapy"[MeSH Terms] |
| #43 | "acupuncture points"[MeSH Terms] |
| #44 | moxibustion[MeSH Terms] |
| #45 | electroacupuncture[MeSH Terms] |
| #46 | "acupuncture, ear"[MeSH Terms] |
| #47 | auriculotherapy[MeSH Terms] |
| #48 | "transcutaneous electric nerve stimulation"[MeSH Terms] |
| #49 | acupressure[MeSH Terms] |
| #50 | #41 OR #42 OR #43 OR #44 OR #45 OR #46 OR #47 OR #48 OR #49 |
| #51 | acupuncture[Title/Abstract] |
| #52 | acupoint*[Title/Abstract] |
| #53 | moxibustion[Title/Abstract] |
| #54 | "auricular acupuncture"[Title/Abstract] |
| #55 | "acupuncture, ear"[Title/Abstract] |
| #56 | auriculotherapy[Title/Abstract] |
| #57 | "auricular point"[Title/Abstract] |
| #58 | "transcutaneous electric nerve stimulation"[Title/Abstract] |
| $59 | "transcutaneous electric acupoint"[Title/Abstract] |
| #60 | electroacupuncture[Title/Abstract] |
| #61 | acupressure[Title/Abstract] |
| #62 | "catgut embedding"[Title/Abstract] |
| #63 | "embedding therap*"[Title/Abstract] |
| #64 | "scalp acupuncture"[Title/Abstract] |
| #65 | #51 #52 OR #53 OR #54 OR #55 OR #56 OR #57 OR #58 OR #59 OR #60 OR #61 OR #62 OR #63 OR #64 |
| #66 | #50 OR #65 |
| #67 | #24 AND #40 AND #66 |

## Table S2 the search strategy of Cochrane

| sequence number | content  retrieval |
| --- | --- |
| #1 | MeSH descriptor: [Neoplasms] explode all trees |
| #2 | (neoplas*):ti,ab,kw |
| #3 | (tumor*):ti,ab,kw |
| #4 | (cancer*):ti,ab,kw |
| #5 | (malignanc*):ti,ab,kw |
| #6 | (oncolog*):ti,ab,kw |
| #7 | (carcino*):ti,ab,kw |
| #8 | (lymphoma):ti,ab,kw |
| #9 | (leukemia):ti,ab,kw |
| #10 | (metastas*):ti,ab,kw |
| #11 | #2 OR #3 OR #4 OR #5 OR #6 OR #7 OR #8 OR #9 OR #10 |
| #12 | #1 OR #11 |
| #13 | MeSH descriptor: [Sleep] explode all trees |
| #14 | MeSH descriptor: [Sleep Initiation and Maintenance Disorders] explode all trees |
| #15 | MeSH descriptor: [Sleep Wake Disorders] explode all trees |
| #16 | MeSH descriptor: [Dyssomnias] explode all trees |
| #17 | MeSH descriptor: [Sleep Disorders, Circadian Rhythm] explode all trees |
| #18 | MeSH descriptor: [Sleep Disorders, Intrinsic] explode all trees |
| #19 | MeSH descriptor: [Sleep Deprivation] explode all trees |
| #20 | MeSH descriptor: [Wakefulness] explode all trees |
| #21 | #13 OR #14 OR #15 OR #16 OR #17 OR #18 OR #19 OR #20 |
| #22 | (Sleep Initiation and Maintenance Disorders):ti,ab,kw |
| #23 | (sleep*):ti,ab,kw |
| #24 | (insomnia):ti,ab,kw |
| #25 | (early waking):ti,ab,kw |
| #26 | dyssomnias):ti,ab,kw |
| #27 | #22 OR #23 OR #24 OR #25 OR #26 |
| #28 | #21 OR #27 |
| #29 | MeSH descriptor:[acupuncture] explore all trees |
| #30 | MeSH descriptor:[acupuncture therapy] explore all trees |
| #31 | MeSH descriptor:[acupuncture points] explore all trees |
| #32 | MeSH descriptor:[moxibustion] explore all trees |
| #33 | MeSH descriptor:[electroacupuncture] explore all trees |
| #34 | MeSH descriptor:[acupuncture, ear] explore all trees |
| #35 | MeSH descriptor:[auriculotherapy] explore all trees |
| #36 | MeSH descriptor: [Acupuncture Points] explode all trees |
| #37 | MeSH descriptor:[transcutaneous electric nerve stimulation] explore all trees |
| #38 | MeSH descriptor:[acupressure] explore all trees |
| #39 | #29 OR #30 OR #31 OR #32 OR #33 OR #34 OR #35 OR #36 OR #37 OR #38 |
| #40 | (acupuncture):ti,ab,kw |
| #41 | (acupoint*):ti,ab,kw |
| #42 | (moxibustion):ti,ab,kw |
| #43 | (auricular acupuncture):ti,ab,kw |
| #44 | (acupuncture, ear):ti,ab,kw |
| #45 | (auriculotherapy):ti,ab,kw |
| #46 | (auricular point):ti,ab,kw |
| #47 | (transcutaneous electric nerve stimulation):ti,ab,kw |
| #48 | (transcutaneous electric acupoint):ti,ab,kw |
| #49 | (electroacupuncture):ti,ab,kw |
| #50 | (acupressure):ti,ab,kw |
| #51 | (catgut embedding):ti,ab,kw |
| #52 | (embedding therap*):ti,ab,kw |
| #53 | (scalp acupuncture):ti,ab,kw |
| #54 | #40 OR #41 OR #42 OR #43 OR #44 OR #45 OR #46 OR #47 OR #48 OR #49 OR #50 OR #51 OR #52 OR #53 |
| #55 | #12 AND #28 AND #54 |

## Table S3 the search strategy of Embase

| sequence number | content  retrieval |
| --- | --- |
| #1 | 'neoplasm'/exp |
| #2 | 'neoplas*':ti,ab,kw |
| #3 | 'tumour*':ti,ab |
| #4 | 'cancer':ti,ab |
| #5 | 'malignanc*':ti,ab |
| #6 | 'oncolog*':ti,ab |
| #7 | 'carcino*':ti,ab,kw |
| #8 | 'lymphoma':ti,ab,kw |
| #9 | 'leukemia':ti,ab,kw |
| #10 | 'metastas*':ti,ab,kw |
| #11 | #2 OR #3 OR #4 OR #5 OR #6 OR #7 OR #8 OR #9 OR #10 |
| #12 | #1 OR #11 |
| #13 | 'insomnia'/exp |
| #14 | 'sleep time'/exp |
| #15 | 'sleep debt'/exp |
| #16 | 'sleep disorder'/exp |
| #17 | 'fragmented sleep'/exp |
| #18 | 'sleep deprivation'/exp |
| #19 | 'sleep quality'/exp |
| #20 | 'sleep disorders, circadian rhythm'/exp |
| #21 | 'wakefulness'/exp |
| #22 | 'circadian rhythm'/exp |
| #23 | #13 OR #14 OR #15 OR #16 OR #17 OR #18 OR #19 OR #20 OR #21 OR #22 |
| #24 | 'sleep initiation and maintenance disorders':ti,ab |
| #25 | 'insomnia':ti,ab |
| #26 | 'sleep*':ti,ab |
| #27 | 'dyssomnias':ti,ab |
| #28 | 'early waking':ti,ab |
| #29 | #24 OR #25 OR #26 OR #27 OR #28 |
| #30 | #23 OR #29 |
| #31 | 'acupuncture'/exp |
| #32 | 'electroacupuncture'/exp |
| #33 | 'transcutaneous electrical nerve stimulation'/exp |
| #34 | 'acupuncture therapy'/exp |
| #35 | 'acupressure'/exp |
| #36 | 'auricular acupuncture'/exp |
| #37 | 'moxibustion'/exp |
| #38 | 'warm acupuncture'/exp |
| #39 | 'catgut embedding'/exp |
| #40 | 'acupuncture point'/exp |
| #41 | #31 OR #32 OR #33 OR #34 OR #35 OR #36 OR #37 OR #38 OR #39 OR #40 |
| #42 | 'acupuncture':ti,ab,kw |
| #43 | 'acupoint*':ti,ab,kw |
| #44 | 'moxibustion':ti,ab,kw |
| #45 | 'warm acupuncture':ti,ab,kw |
| #46 | 'auricular acupuncture':ti,ab,kw |
| #47 | 'acupuncture, ear':ti,ab,kw |
| #48 | 'auriculotherapy':ti,ab,kw |
| #49 | 'auricular point':ti,ab,kw |
| #50 | 'transcutaneous electric nerve stimulation':ti,ab,kw |
| #51 | 'transcutaneous electric acupoint':ti,ab,kw |
| #52 | 'electroacupuncture':ti,ab,kw |
| #53 | 'acupressure':ti,ab,kw |
| #54 | 'catgut embedding':ti,ab,kw |
| #55 | 'embedding therap*':ti,ab,kw |
| #56 | 'scalp acupuncture':ti,ab,kw |
| #57 | #42 OR #43 OR #44 OR #45 OR #46 OR #47 OR #48 OR #49 OR #50 OR #51 OR #52 OR #53 OR #54 OR #55 OR #56 |
| #58 | #41 OR #57 |
| #59 | #12 AND #30 AND #58 |

## Table S4 the search strategy of Web of Science

| sequence number | content  retrieval |
| --- | --- |
| #1 | Topic: (neoplas*) |
| #2 | Topic: (tumor*) |
| #3 | Topic: (cancer*) |
| #4 | Topic: (malignanc*) |
| #5 | Topic: (oncolog*) |
| #6 | Topic: (Lymphoma) |
| #7 | Topic: (metastas*) |
| #8 | Topic: (carcino*) |
| #9 | Topic: (Leukemia) |
| #10 | #1 OR #2 OR #3 OR #4 OR #5 OR #6 OR #7 OR #8 OR #9 |
| #11 | Topic: (Sleep Initiation and Maintenance Disorders) |
| #12 | Topic: (insomnia) |
| #13 | Topic: (early waking) |
| #14 | Topic: (Dyssomnias) |
| #15 | Topic: (sleep*) |
| #16 | #11 OR #12 OR#13 OR #14 OR #15 |
| #17 | Topic: (acupuncture) |
| #18 | Topic: (acupoint) |
| #19 | Topic: (moxibustion) |
| #20 | Topic: (acupuncture, ear) |
| #21 | Topic: (auricular acupuncture) |
| #22 | Topic: (auriculotherapy) |
| #23 | Topic: (transcutaneous electric nerve stimulation) |
| #24 | Topic: (transcutaneous electric acupoint) |
| #25 | Topic: (electroacupuncture) |
| #26 | Topic: (acupressure) |
| #27 | Topic: (catgut embedding) |
| #28 | Topic: (embedding therap*) |
| #29 | Topic: (acupuncture therapy) |
| #30 | Topic: (acupuncture points) |
| #31 | Topic: (scalp acupuncture) |
| #32 | #17 OR 18 OR #19 OR #20 OR #21 OR #22 OR #23 OR #24 OR #25 OR #26 OR #27 OR #28 OR #29 OR #30 OR #31 |
| #33 | #10 AND #16 AND #32 |

## Table S5 the search strategy of CBM

| sequence number | content  retrieval |
| --- | --- |
| #1 | “neoplasm” [Weighted: extended] |
| #2 | “neoplasm” [Common field: Intelligence] |
| #3 | “tumor” [Common field: Intelligence] |
| #4 | “cancer” [Common field: Intelligence] |
| #5 | “Hodgkin's disease” [Common field: Intelligence] |
| #6 | “leukemia” [Common field: Intelligence] |
| #7 | #2 OR #3 OR #4 OR # 5 OR #6 |
| #8 | #1 OR #7 |
| #9 | “Sleep Initiation and Maintenance Disorders” [Weighted: extended] |
| #10 | “insomnia” [Weighted: extended] |
| #11 | #9 OR #10 |
| #12 | “insomnia” [Common field: Intelligence] |
| #13 | “Sleep Initiation and Maintenance Disorders” [Common field: Intelligence] |
| #14 | “sleep” [Common field: Intelligence] |
| #15 | “early waking” [Common field: Intelligence] |
| #16 | “dreaminess” [Common field: Intelligence] |
| #17 | #12 OR #13 OR #14 OR # 15 OR #16 |
| #18 | #11 OR #17 |
| #19 | “acupuncture” [Weighted: extended] |
| #20 | “acupuncture therapy” [Weighted: extended] |
| #21 | “moxibustion” [Weighted: extended] |
| #22 | “needle warming therapy” [Weighted: extended] |
| #23 | “thunderbolt moxibustion therapy” [Weighted: extended] |
| #24 | “electroacupuncture” [Weighted: extended] |
| #25 | “auriculotherapy” [Weighted: extended] |
| #26 | “acupuncture, ear” [Weighted: extended] |
| #27 | “auricular acupuncture therapy” [Weighted: extended] |
| #28 | “auricular plaster therapy” [Weighted: extended] |
| #29 | “intradermal needle therapy” [Weighted: extended] |
| #30 | “scalp acupuncture therapy” [Weighted: extended] |
| #31 | “acupoint therapy” [Weighted: extended] |
| #32 | “acupressure” [Weighted: extended] |
| #33 | “acupoint” [Weighted: extended] |
| #34 | “transcutaneous electric nerve stimulation Therapy” [Weighted: extended] |
| #35 | “acupoint sticking therapy” [Weighted: extended] |
| #36 | #19 OR #20 OR #21 OR #22 OR #23 OR #24 OR # 25 OR #26 OR #27 OR #28 OR #29 OR #30 OR #31 OR #32 OR #33 OR #34 OR #35 |
| #37 | “acupuncture” [Common field: Intelligence] |
| #38 | “acupuncture therapy” [Common field: Intelligence] |
| #39 | “moxibustion” [Common field: Intelligence] |
| #40 | “needle warming therapy” [Common field: Intelligence] |
| #41 | “thunderbolt moxibustion therapy” [Common field: Intelligence] |
| #42 | “electroacupuncture” [Common field: Intelligence] |
| #43 | “auriculotherapy” [Common field: Intelligence] |
| #44 | “acupuncture, ear” [Common field: Intelligence] |
| #45 | “auricular acupuncture therapy” [Common field: Intelligence] |
| #46 | “auricular plaster therapy” [Common field: Intelligence] |
| #47 | “intradermal needle therapy” [Common field: Intelligence] |
| #48 | “scalp acupuncture therapy” [Common field: Intelligence] |
| #49 | “acupoint therapy” [Common field: Intelligence] |
| #50 | “acupressure” [Common field: Intelligence] |
| #51 | “acupoint” [Common field: Intelligence] |
| #52 | “transcutaneous electric nerve stimulation Therapy” [Common field: Intelligence] |
| #53 | “transcutaneous electric acupoint stimulation Therapy” [Common field: Intelligence] |
| #54 | “TEAS” [Common field: Intelligence] |
| #55 | “TENS” [Common field: Intelligence] |
| #56 | “acupoint sticking therapy” [Common field: Intelligence] |
| #57 | “embedding therapy” [Common field: Intelligence] |
| #58 | #37 OR #38 OR #39 OR #40 OR #41 OR #42 OR #43 OR #44 OR #45 OR #46 OR #47 OR #48 OR #49 OR #50 OR #51 OR #52 OR #53 OR #54 OR #55 OR #56 OR #57 |
| #59 | #36 OR #58 |
| #60 | #8 AND #18 AND #59 |

## Table S6 the search strategy of VIP

| sequence number | content  retrieval |
| --- | --- |
| #1 | M=neoplasm |
| #2 | M=tumor |
| #3 | M=cancer |
| #4 | M=Hodgkin's disease |
| #5 | M=leukemia |
| #6 | #1 OR #2 OR #3 OR #4 OR #5 |
| #7 | M=insomnia |
| #8 | M=Sleep Initiation and Maintenance Disorders |
| #9 | M=sleep |
| #10 | M=early waking |
| #11 | M=dreaminess |
| #12 | #7 OR #8 OR #9 OR #10 OR #11 |
| #13 | M=acupuncture |
| #14 | M=acupuncture therapy |
| #15 | M=moxibustion |
| #16 | M=needle warming therapy |
| #17 | M=thunderbolt moxibustion therapy |
| #18 | M=electroacupuncture |
| #19 | M=auriculotherapy |
| #20 | M=acupuncture, ear |
| #21 | M=auricular acupuncture therapy |
| #22 | M=auricular plaster therapy |
| #23 | M=intradermal needle therapy |
| #24 | M=scalp acupuncture therapy |
| #25 | M=acupoint therapy |
| #26 | M=acupressure |
| #27 | M=acupoint |
| #28 | M=transcutaneous electric nerve stimulation Therapy |
| #29 | M=transcutaneous electric acupoint stimulation Therapy |
| #30 | M=TEAS |
| #31 | M=TENS |
| #32 | M=acupoint sticking therapy |
| #33 | M=embedding therapy |
| #34 | #13 OR #14 OR # 15 OR #16 OR #17 OR #18 OR #19 OR #20 OR #21 OR #22 OR #23 OR #24 OR # 25 OR #26 OR #27 OR #28 OR #29 OR #30 OR #31 OR #32 OR #33 |
| #35 | #6 AND #12 AND #34 |

## Table S7 the search strategy of Wanfang Database

| sequence number | content  retrieval |
| --- | --- |
| #1 | Subject:(neoplasm) |
| #2 | Subject:(tumor) |
| #3 | Subject:(cancer) |
| #4 | Subject:(Hodgkin's disease) |
| #5 | Subject:(leukemia) |
| #6 | #1 OR #2 OR #3 OR #4 OR #5 |
| #7 | Subject:(insomnia) |
| #8 | Subject:(Sleep Initiation and Maintenance Disorders) |
| #9 | Subject:(sleep) |
| #10 | Subject:(early waking) |
| #11 | Subject:(dreaminess) |
| #12 | #7 OR #8 OR #9 OR #10 OR #11 |
| #13 | Subject:(acupuncture) |
| #14 | Subject:(acupuncture therapy) |
| #15 | Subject:(moxibustion) |
| #16 | Subject:(needle warming therapy) |
| #17 | Subject:(thunderbolt moxibustion therapy) |
| #18 | Subject:(electroacupuncture) |
| #19 | Subject:(auriculotherapy) |
| #20 | Subject:(acupuncture, ear) |
| #21 | Subject:(auricular acupuncture therapy) |
| #22 | Subject:(auricular plaster therapy) |
| #23 | Subject:(intradermal needle therapy) |
| #24 | Subject:(scalp acupuncture Therapy) |
| #25 | Subject:(acupoint therapy) |
| #26 | Subject:(acupressure) |
| #27 | Subject:(acupoint) |
| #28 | Subject:(transcutaneous electric nerve stimulation Therapy) |
| #29 | Subject:(transcutaneous electric acupoint stimulation Therapy) |
| #30 | Subject:(TEAS) |
| #31 | Subject:(TENS) |
| #32 | Subject:(acupoint sticking therapy) |
| #33 | Subject:(embedding therapy) |
| #34 | #13 OR #14 OR # 15 OR #16 OR #17 OR #18 OR #19 OR #20 OR #21 OR #22 OR #23 OR #24 OR # 25 OR #26 OR #27 OR #28 OR #29 OR #30 OR #31 OR #32 OR #33 |
| #35 | #6 AND #12 AND #34 |

## Table S8 the search strategy of CNKI

| sequence number | content  retrieval |
| --- | --- |
| #1 | ti,kw,ab=neoplasm |
| #2 | ti,kw,ab=tumor |
| #3 | ti,kw,ab=cancer |
| #4 | ti,kw,ab=Hodgkin's disease |
| #5 | ti,kw,ab=leukemia |
| #6 | #1 OR #2 OR #3 OR #4 OR #5 |
| #7 | ti,kw,ab=insomnia |
| #8 | ti,kw,ab=Sleep Initiation and Maintenance Disorders |
| #9 | ti,kw,ab=sleep |
| #10 | ti,kw,ab=early waking |
| #11 | ti,kw,ab=dreaminess |
| #12 | #7 OR #8 OR #9 OR #10 OR #11 |
| #13 | ti,kw,ab=acupuncture |
| #14 | ti,kw,ab=acupuncture therapy |
| #15 | ti,kw,ab=moxibustion |
| #16 | ti,kw,ab=needle warming therapy |
| #17 | ti,kw,ab=thunderbolt moxibustion therapy |
| #18 | ti,kw,ab=electroacupuncture |
| #19 | ti,kw,ab=auriculotherapy |
| #20 | ti,kw,ab=acupuncture, ear |
| #21 | ti,kw,ab=auricular acupuncture therapy |
| #22 | ti,kw,ab=auricular plaster therapy |
| #23 | ti,kw,ab=intradermal needle therapy |
| #24 | ti,kw,ab=scalp acupuncture therapy |
| #25 | ti,kw,ab=acupoint therapy |
| #26 | ti,kw,ab=acupressure |
| #27 | ti,kw,ab=acupoint |
| #28 | ti,kw,ab=transcutaneous electric nerve stimulation Therapy |
| #29 | ti,kw,ab=transcutaneous electric acupoint stimulation Therapy |
| #30 | ti,kw,ab=TEAS |
| #31 | ti,kw,ab=TENS |
| #32 | ti,kw,ab=acupoint sticking therapy |
| #33 | ti,kw,ab=embedding therapy |
| #34 | #13 OR #14 OR # 15 OR #16 OR #17 OR #19 OR #20 OR #21 OR #22 OR #23 OR #24 OR # 25 OR #26 OR #27 OR #28 OR #29 OR #30 OR #31 OR #32 OR #33 |
| #35 | #6 AND #12 AND #34 |

# Part 7 The usage of acupoints in included studies

## Table S9 the usage of acupoints in included studies

| Acupoints | Frequency of Usage |
| --- | --- |
| Xin (heart, CO15) | 20 |
| Shenmen (TF4) | 19 |
| Pizhixia (subcortex, AT4) | 14 |
| HT7, Jiaogan (sympathetic, AH6a) | 13 |
| SP6 | 12 |
| PC6 | 10 |
| ST36, GV20, EX-HN3, EX-HN1, Shen (kidney, CO10) | 9 |
| Gan (liver, CO12) | 8 |
| KI1 | 7 |
| GV24, Pi (spleen, CO13) | 6 |
| Neifenmi (CO18), BL15 | 5 |
| BL23, RN8, RN4 | 4 |
| RN6, BL20, Fei (lung, CO14), KI3 | 3 |
| Shenshangxian (TG2p), Wei (stomach, CO4), SJ5, BL62, LR3, PC8, GV3, RN12, Anmian | 2 |
| Nangan(brainstem, AT3,4i), zhen(AT3), Dachang(colon, CO7), Dachang (CO7), Chuiqian (LO4), GB17, GV22, RN10, GB15, EX-HN5, SP3, RN9, LI11, LI4, EX-HN6, BL19, GB20, ST8, RN17, SP10, BL63, KI4, SP4, GB41, SI3, LU7, KI6 | 1 |
